# Exome sequencing of Pakistani consanguineous families identifies 30 novel candidate genes for recessive intellectual disability

**DOI:** 10.1038/mp.2016.109

**Published:** 2016-07-26

**Authors:** S Riazuddin, M Hussain, A Razzaq, Z Iqbal, M Shahzad, D L Polla, Y Song, E van Beusekom, A A Khan, L Tomas-Roca, M Rashid, M Y Zahoor, W M Wissink-Lindhout, M A R Basra, M Ansar, Z Agha, K van Heeswijk, F Rasheed, M Van de Vorst, J A Veltman, C Gilissen, J Akram, T Kleefstra, M Z Assir, UK10K, D Grozeva, K Carss, F L Raymond, T D O'Connor, S A Riazuddin, S N Khan, Z M Ahmed, A P M de Brouwer, H van Bokhoven, S Riazuddin

**Affiliations:** 1 Department of Otorhinolaryngology—Head and Neck Surgery, University of Maryland, School of Medicine, Baltimore, MD USA; 2 Center for Genetic Diseases, Shaheed Zulfiqar Ali Bhutto Medical University, Pakistan Institute of Medical Sciences, Islamabad, Pakistan; 3 Department of Human Genetics, Donders Institute for Brain, Cognition and Behaviour, Radboud University Medical Center, Nijmegen, The Netherlands; 4 Allama Iqbal Medical College, University of Health Sciences, Lahore, Pakistan; 5 National Centre of Excellence in Molecular Biology, University of The Punjab, Lahore, Pakistan; 6 Center for Genetic Diseases, CAPES Foundation, Ministry of Education of Brazil, Brasília, Brazil; 7 Institute for Genome Sciences and Program in Personalized and Genomic Medicine, University of Maryland, School of Medicine, Baltimore, MD USA; 8 Department of Biosciences, Faculty of Science, COMSATS Institute of Information Technology, Islamabad, Pakistan; 9 Department of Clinical Genetics, GROW School for Oncology and Developmental Biology, Maastricht University Medical Centre, Maastricht, The Netherlands; 10 The Wellcome Trust Sanger Institute, Wellcome Trust Genome Campus, Cambridge UK; 11 Department of Medical Genetics, Cambridge Institute for Medical Research, University of Cambridge, Cambridge, UK; 12 Department of Haematology, University of Cambridge, Cambridge, UK; 13 Department of Ophthalmology, The Wilmer Eye Institute, Johns Hopkins University School of Medicine, Baltimore, MD USA; 14 Present Address: 14Current address: Department of Neurology, Oslo University Hospital, Oslo, Norway., ,; 15 Present Address: 15Current address: Department of Genetic Medicine and Development, University of Geneva, Geneva, Switzerland., ,

**Keywords:** Psychiatric disorders, Genetics

## Abstract

Intellectual disability (ID) is a clinically and genetically heterogeneous disorder, affecting 1–3% of the general population. Although research into the genetic causes of ID has recently gained momentum, identification of pathogenic mutations that cause autosomal recessive ID (ARID) has lagged behind, predominantly due to non-availability of sizeable families. Here we present the results of exome sequencing in 121 large consanguineous Pakistani ID families. In 60 families, we identified homozygous or compound heterozygous DNA variants in a single gene, 30 affecting reported ID genes and 30 affecting novel candidate ID genes. Potential pathogenicity of these alleles was supported by co-segregation with the phenotype, low frequency in control populations and the application of stringent bioinformatics analyses. In another eight families segregation of multiple pathogenic variants was observed, affecting 19 genes that were either known or are novel candidates for ID. Transcriptome profiles of normal human brain tissues showed that the novel candidate ID genes formed a network significantly enriched for transcriptional co-expression (*P<*0.0001) in the frontal cortex during fetal development and in the temporal–parietal and sub-cortex during infancy through adulthood. In addition, proteins encoded by 12 novel ID genes directly interact with previously reported ID proteins in six known pathways essential for cognitive function (*P<*0.0001). These results suggest that disruptions of temporal parietal and sub-cortical neurogenesis during infancy are critical to the pathophysiology of ID. These findings further expand the existing repertoire of genes involved in ARID, and provide new insights into the molecular mechanisms and the transcriptome map of ID.

**Supplementary information:**

The online version of this article (doi:10.1038/mp.2016.109) contains supplementary material, which is available to authorized users.

## Introduction

Intellectual disability (ID) is a common neurodevelopmental disorder with an onset of cognitive impairment before the age of 18 years^1, 2, 3^ and is characterized by significant limitations in intellectual functioning and adaptive behavior.^1^ The disease affects 1–3% of the world population; however, its prevalence in the developing world is almost twice that of the affluent world.^4, 5^ The causes of nearly 40% of ID remain ambiguous.^6^ Among the known causes, ~50% of ID cases have an environmental etiology such as poor nutrition, multiple pregnancies with little gap, prenatal/perinatal brain ischemia, postnatal infections and inadequate medical services. The other half of ID cases has a genetic etiology, such as chromosomal abnormalities or mutations in specific genes.^3, 5^

In the western world, *de novo* heterozygous mutations and genomic copy number changes account for the majority of ID cases.^7^ In contrast, recessive ID appears to be more common in consanguineous populations. Identification of gene mutations that cause non-syndromic autosomal recessive ID (ARID) has been notoriously slow because of the scarcity of sizeable families. By 2006, only three genes, *CC2D1A*, *CRBN* and *PRSS12* had been associated with ARID.^8, 9, 10^ After 2006, research studies involving highly inbred populations from North Africa, the Middle East and South East Asia, greatly accelerated the pace of identification of gene mutations that cause ARID. These studies were further augmented by the use of next-generation sequencing. In 2011, Najmabadi *et al.*^11^ applied targeted next-generation sequencing to 136 consanguineous Iranian families, in which homozygosity mapping had previously identified a locus, and reported 23 known and 50 new candidate ARID genes. More recently, exome sequencing (ES) of 143 large consanguineous Saudi Arabian families revealed 33 novel candidate genes involved in different neurological disorders.^12^ These studies further highlighted the clinical and genetic complexity of ID and other neurodevelopmental disorders. Intriguingly, not a single gene with pathogenic variants emerged across various populations. Despite this progress, a large number of potential pathogenic gene mutations remain unidentified and warrant further studies in extended families from communities with endogamy.

Consanguineous marriages are practiced by about 20% of the human population, and the extent of consanguinity varies among different citizenry in the world. The rate of consanguineous marriages is 38% in Iran,^13, 14^ over 40% in several Middle Eastern countries^15^ and above 50% in Pakistan.^16^ The elevated level of endogamy in Pakistan has led to the increased prevalence of genetic disorders, including ARID, with an average of 1.1 cases of severe ID and 6.2 cases of mild ID per 100 live births.^17^ The present study is designed to identify pathogenic gene mutations that cause ARID in the highly inbred population of Pakistan. In all, 121 families of 8 different ethnicities, exhibiting recessive ID, were enrolled mostly from the rural population of Pakistan. Through a systematic exome-sequencing approach we have identified potential pathogenic variants in 68 of these families: 30 families with a single homozygous DNA variant affecting previously known ID genes and another 30 families with a single homozygous or compound heterozygous variants in novel candidate ID genes. In eight families multiple homozygous variants were identified. We report the phenotype–genotype relationships, the predicted pathogenicity of the newly discovered candidate genes, their co-expression in functional networks in the developing and adult human brain, and possible involvement in various cellular processes.

## Materials and methods

### Family ascertainment and phenotype analysis

This study was approved by the Institutional Review Board of the Centre of Excellence in Molecular Biology (CEMB), University of the Punjab, Lahore, Pakistan and the Medical Ethical Committee Arnhem-Nijmegen, The Netherlands. The families of our cohort were obtained mainly from the rural populations of all five provinces of Pakistan (Supplementary Tables S1 and S2). Written informed consents were obtained from healthy adult subjects and the parents/legal guardians of minor subjects and ID patients. Specific informed consent was obtained for showing images of patients (Supplementary Figure S1). Participating individuals were evaluated with medical history interviews, and ID-related phenotypic features of patients were recorded. All affected individuals were clinically evaluated by both a geneticist and a general medical practitioner, with particular attention to neurological, morphological, ophthalmological, dermatological and skeletal symptoms. Photographs of the face and uncovered limbs were also taken (Supplementary Figure S1). In some cases, magnetic resonance imaging and computerized tomography scans were also obtained. A description of developmental milestones was used to evaluate the severity of ID.^18^ Only families with confirmed existence of cognitive dysfunction in the affected members were included in this study. Peripheral blood samples were collected from participating subjects. Genomic DNA was isolated following standard procedures.^19^

### Exome sequencing and variant selection

Exome enrichment and high-throughput sequencing were performed at the Radboudumc (Nijmegen, The Netherlands), The Wellcome Trust Sanger Institute (Hinxton, as part of the UK10K study) and the University of Maryland as previously described.^20^ Selection of high-quality, potentially pathogenic variants was performed using seven major filtration steps (Supplementary Figure S2 and Supplementary Methods).

### Sanger sequencing

ES results were confirmed by Sanger sequencing. Primers for the amplification of the exons carrying variants were designed by using Primer3 (ref. 21; Supplementary Tables S9 and S10).

### Predicted involvement of candidate genes in cellular processes and pathways

We researched the available literature and extracted all reported protein–protein interactions involving the known and the newly identified ID proteins and then entered them into a set of protein–protein interaction networks via the STRING^22^ and MATISSE^23^ algorithms, which are included in the EXPANDER package tools.^24^

### Expression of candidate genes in the human brain

Brain expression analyses for new ARID genes were conducted using existing data sets in the BrainSpan: Atlas of the Developing Human Brain.^25^ Normalized gene expression levels for 26 different brain tissues from 31 different developmental periods were obtained from the BrainSpan RNA-seq data set v3 (http://brainspan.org) of the developing human brain.

### Co-expression network analysis

Networks of functionally co-expressed genes were identified by using the model of Gulsuner *et al.*^26^ Using the BrainSpan database, gene pairs are defined as connected if the absolute value of the Pearson’s correlation coefficient is >0.8 for their expression levels in different brain regions (frontal cortex, temporal-parietal, sensory–motor and sub-cortical) and different developmental stages (fetal, infancy to late childhood and adolescence to adulthood), totaling 12 different networks. For each of these networks the numbers of edges (connections) between each of the novel ID genes was calculated. Random simulations of 10 000 replicates of an equal number of genes randomly selected from the BrainScan database were conducted to establish the significance of the connections. This provided a distribution of edges to estimate an empirical *P*-value for our novel ID genes.

In addition, we also randomly selected genes from the BrainScan database with an HGNC gene symbol, as well as at least one connection in the temporal region network. We compared an equal number of genes in this set with our ID genes that passed the same filtering criterion, which provided more conservative and consistent *P*-values.

## Results

### Recruitment of families

We enrolled a cohort of 121 ID families with a likely autosomal recessive inheritance pattern mostly from the rural areas of Punjab, Sindh, Baluchistan, Khyber Pakhtoon Khawa and Northern areas. The enrolled families comprise eight ethnic groups, namely Punjabi (68.6%), Siraiki (10.8%), Pathan (9%), Urdu speaking (7.8%) and others (Sindhi, Afghan, Baloch and Kashmiri (3.8%); Supplementary Table S1). Every family had two or more affected individuals (except PKMR51a) and 111 families had consanguineous unions, while in 74 families the affected individuals were present in separate sibships (Supplementary Table S2).

### Genetic analysis

Before ES, the presence of disease-causing copy number variations was excluded by SNP microarray analysis (Affymetrix 250 K SNP array or higher coverage). ES was carried out for DNA samples of 1–3 individuals per family. Selection of the nucleotide variants was performed using the seven-tier filtering strategy (Supplementary Figure S2). Sanger sequencing confirmed 359 candidate pathogenic variants. Of these, 80 variants in 77 genes segregated with the ID phenotype. There are six categories of evidence that we are using in this study to support an assertion that a variant is potentially pathogenic. These are (1) segregation analysis; (2) either absence in the dbSNP142 database or a very low allele frequency (<0.01) in the ExAC Browser database; (3) absence in 213 ethnically matched control individuals; (4) a CADD score >20; (5) pathogenicity prediction by multiple *in silico* programs; and (6) enrichment of loss-of-function (LOF) and potentially pathogenic variants of known and novel ID genes in patients with ID as compared with individuals with the non-ID phenotype (*n*=213; Supplementary Table S3).

### Pathogenic variants identified in previously reported ARID genes

We identified 34 predicted pathogenic variants in 32 genes previously associated with ID or related neurodevelopmental disorders (Tables 1 and 3a; Supplementary Figures S3 and S6). These variants include homozygous missense (*n*=21), nonsense (*n*=5), frameshift (*n*=5) and splice site variants (*n*=3). Twenty-five of these variants are novel (Tables 1 and 3a). All new variants segregated with the disease and were either absent in dbSNP142 or had an allele frequency ⩽0.002 in the ExAC Browser^27^ (Tables 1 and 3a).

**Table 1:** Variants identified in known genes for intellectual disability or related disorders

*Family*	*Gene*	*Position* ^a^	*Transcript*	*cDNA mutation* ^b^	*Protein change*	*Variation*	*CADD score v1.3*	*ExAC allele frequency x10* ^− *6*^	*OMIM*	*Phenotype* ^c^
PKMR08	*GNE*	Chr9:36217445C>T	NM_005476.5	c.2086G>A	p.(Val696Met)	Missense	32.0	1942	603824	D
PKMR29	*POMT2*	Chr14:77765840T>C	NM_013382.5	c.881A>G	p.(Tyr294Cys)	Missense	27.9	0	607439	O
PKMR36	*APTX*	Chr9:32984803delC	NM_001195248.1	c.638delG	p.(Arg213Leufs*15)	Frameshift	NA	8.238	606350	O
PKMR42	*VPS13B*	Chr8:100654333C>T	NM_017890.4	c.5590C>T	p.(Gln1864*)	Nonsense	NA	0	607817	O
PKMR51a	*TSHR*	Chr14:81610687C>T	NM_000369.2	**c.2285C>T**	**p.(Thr762Met)**	Missense	25.3	8.247	603372	D
PKMR61a	*SCN1A*	Chr2:166848284G>A	NM_001165963.1	c.5501C>T	p.(Ala1834Val)	Missense	32	8.241	182389^d^	O
PKMR79	*AP4M1*	Chr7:99704430delG	NM_004722.3	**c.1287delG**	p.(Arg429Serfs*15)	Frameshift	NA	0	602296	O
PKMR82	*KCNA2*	Chr1:111147212G>A	NM_004974.3	**c.193C>T**	p.(Arg65*)	Nonsense	NA	8.239	176262^d^	C, inheritance D
PKMR85	*MED23*	Chr6:131941859T>C	NM_004830.3	**c.506A>G**	**p.(Tyr169Cys)**	Missense	24.6	0	605042	C
PKMR86	*FRAS1*	Chr4:79202579C>T	NM_025074.6	**c.1099C>T**	**p.(Arg367Cys)**	Missense	24.4	43.14	607830	O
PKMR87	*MAN2B1*	Chr19:12763176C>G	NM_000528.3	**c.1928+1C>G**	p.(Phe642Phefs*2)	Aberrant splicing	NA	0	609458	O
PKMR97	*MFSD2A*	Chr1:40431565C>T	NM_001136493.2	**c.632C>T**	**p.(Thr211Met)**	Missense	33	8.239	614397	C
PKMR99	*SYNE1*	Chr6:152819877C>G	NM_182961.3	**c.939G>C**	**p.(Lys313Asn)**	Missense	21.9	28.33	608441	D
PKMR102	*ASPM*	Chr1:197087007C>T	NM_018136.4	**c.3977G>A**	p.(Trp1326*)	Nonsense	NA	8.255	605481	C
PKMR105	*ZNF41*	ChrX:47315776C>T	NM_007130.2	**c.94G>A**	**p.(Val32Met)**	Missense	30	0	314995	O
PKMR115	*SRD5A3*	Chr4:56212560G>A	NM_024592.4	c.57G>A	p.Trp19*	Nonsense	NA	357.1	611715	C
PKMR119	*PGAP1*	Chr2:197710616T>C	NM_024989.3	**c.2276A>G**	**p.(Tyr759Cys)**	Missense	23.6	16.57	611655	C
PKMR133	*DOCK8*	Chr9:286599G>A	NM_203447.3	**c.295G>A**	**p.(Glu99Lys)**	Missense	25.9	247.3	611432	C
PKMR151	*TMEM67*	Chr8:94768056G>A	NM_153704.5	c.274G>A	p.(Gly92Arg)	Missense	25.6	17.41	609884	O
PKMR152	*WDR62*	Chr19:36587982G>T	NM_001083961.1	**c.2520+1G>T**	p.(Leu840Leufs*95)	Aberrant splicing	NA	0	613583	C
PKMR184	*SPG11*	Chr15:44876109delA	NM_025137.3	c.5769delT	p.(Ser1923Argfs*28)	Frameshift	NA	41.18	610844	C
PKMR188	*ASPA*	Chr17:3402260G>A	NM_000049.2	c.820G>A	p.(Gly274Arg)	Missense	28.9	8.421	608034	C
PKMR193	*ARL13B*	Chr3:93755508G>A	NM_182896.2	**c.599G>A**	**p.(Arg200His)**	Missense	34	8.314	608922	C
PKMR212	*ZFYVE26*	Chr14:68268804_05delGA	NM_015346.3	**c.1630_1631delTC**	p.(Ser544Leufs*24)	Frameshift	NA	8.236	612012	C
PKMR216	*AP4S1*	Chr14:31539047A>G	NM_007077.4	**c.139-2A>G**	p.(Gln46Glnfs*85)	Aberrant splicing	NA	24.71	607243	C
PKMR224	*MKKS*	Chr20:10393388delT	NM_018848.3	**c.775delA**	p.(Thr259Leufs*21)	Frameshift	NA	74.18	604896	C
PKMR242	*WDR73*	Chr15:85186864A>G	NM_032856.3	**c.974T>C**	**p.(Phe325Ser)**	Missense	29.9	0	616144	O
PKMR264	*FRY*	Chr13:32747633G>A	NM_023037.2	**c.2281G>A**	**p.(Val761Ile)**	Missense	21.7	8.281	614818	O
PKMR281	*GPT2*	Chr16:46956326C>T	NM_133443.3	**c.1210C>T**	p.(Arg404*)	Nonsense	NA	33.31	138210	O
PKMR321	*FLNA*	ChrX:153583356G>A	NM_001110556.1	**c.5054C>T**	**p.Thr1685Met**	Missense	27.1	11.58	300017	C

Abbreviations: C, concordant; D, discordant; NA, not applicable; O, overlapping features.

^a^Chromosomal position according to GRCh37/hg19.

^b^Novel mutations are written in bold font.

^c^Concordance of the observed phenotype with the phenotype reported in Online Mendelian Inheritance in Man (OMIM).

^d^Reported phenotypes show a dominant inheritance pattern, whereas the mutations reported here are homozygous.

Most genes were found to be mutated in only one family, suggesting a low incidence of founder mutations underlying ID in the Pakistani population. Two genes, *TMEM67* and *FRAS1*, which carried different variants in two independent families (Tables 1 and 3a). Two families (PKMR61b and PKMR69) carry one homozygous variant each in two unrelated but known ID genes. PKMR61b has variants in *TMEM67* and *FGFR1* while PKMR69 has variants in *FRAS1* and *EXOSC8* (Table 3a; Supplementary Figure S6). No carriers of these variants, which were both predicted to be damaging and disease causing, were detected in our population-specific controls. Two families each harbored a variant in an X-linked gene (*ZNF41* and *FLNA*).

A comparison of the phenotypes of 34 variants in genes known to cause ID shows that in most families the phenotype is similar to the reported entity (Supplementary Table S4). Some deviations do occur, which may result from variable effects of allelic mutations or the effects of genetic modifier variants, which may be particularly relevant in populations with a high consanguinity rate.^28, 29^

### Pathogenic variants identified in novel candidate ARID genes

In 30 families we identified plausible pathogenic variants affecting a single gene that had not been previously implicated in ARID (Table 2; Supplementary Figure S4). The majority of these changes were missense variants (*n*=23). In addition, we identified frameshift (*n*=6), nonsense (*n*=1) and splice site (*n*=1) variants that result in a truncated protein or are predicted to result in nonsense-mediated RNA decay. None of the variants were found in 213 unrelated, ethnically matched control individuals, indicating that they are not indigenous Pakistani polymorphisms. Clustal W (http://www.ebi.ac.uk/Tools/msa/clustalw2/) alignment of protein sequences encompassing the missense variants showed that most of the mutated amino acids are evolutionarily conserved (Supplementary Figure S5). In 9 of the families there were no notable clinical characteristics besides ID, whereas 21 families exhibited syndromic forms of ID (Supplementary Table S5).

**Table 2:** Novel candidate genes for intellectual disability

*Family*	*Ethnicity*	*Genes*	*Position* ^a^	*Transcript*	*cDNA mutation*	*Protein change*	*Variation*	*Protein function*	*CADD score v1.3*	*ExAC allele frequency x10* ^− *6*^
PKMR24	Punjabi	*ZSCAN25 (ZNF498)*	Chr7:99219114A>G	NM_145115.2	c.506A>G	p.(Glu169Gly)	Missense	Zinc finger protein nucleic acid binding	26.5	0
PKMR33	Punjabi	*DPH1*	Chr17:1944817C>T	NM_001383.3	c.1144C>T	p.(Pro382Ser)	Missense	Diphthamide biosynthesis	32	8.515
PKMR40	Pathan	*DCTN2*	Chr12:57929561T>G	NM_001261412.1	c.173A>C	p.(Lys58Thr)	Missense	Structural protein	26.8	8.484
PKMR43	Pathan	*METTL5*	Chr2:170677663_64delTC	NM_014168.3	c.344_345delGA	p.(Arg115Asnfs*19)	Frameshift	Methyl transferase	NA	0
PKMR45	Pathan	*TANGO2 (C22orf25)*	Chr22:20041047G>T	NM_152906.5	c.353G>T	p.(Gly118Val)	Missense	Transport and Golgi organization	29.4	0
PKMR64	Punjabi	*CAPN12* ^b^	Chr19:39230761_62delTT	NM_144691.4	c.658_659delAA	p.(Asn220Glnfs*25)	Frameshift	Cystine protease	NA	795.1
PKMR66	Punjabi	*TBC1D8*	Chr2:101652537G>A	NM_001102426.1	c.1501C>T	p.(Leu501Phe)	Missense	G-protein modulator	31.0	99.37
PKMR67	Punjabi	*MSS51* *(ZMYND17)*	Chr10:75187870G>A	NM_001024593.1	c.173C>T	p.(Ser58Leu)	Missense	Zinc finger transcription factor	33.0	140.1
PKMR72	Punjabi	*MDGA2*	Chr14:47343402T>C	NM_001113498.2	c.2232A>G	p.(Arg744Arg)	Aberrant Splicing	GPI anchor	NA	52.83
PKMR98	Punjabi	*FMOD*	Chr1:203316893C>T	NM_002023.4	c.506G>A	p.(Arg169Gln)	Missense	Receptor	23.3	296.6
PKMR118	Punjabi	*C22orf31*	Chr22:29454885C>G	NM_015370.1	c.718G>C	p.(Gly240Arg)	Missense	Unknown	28.6	0
PKMR142	Punjabi	*SMARCA1*	ChrX:128657269C>T	NM_139035.2	c.79G>A	p.(Glu27Lys)	Missense	Component of NURF complex	22.1	35.55
PKMR153	Punjabi	*GPAA1*	Chr8:145138854G>C	NM_003801.3	c.527G>C	p.(Trp176Ser)	Missense	Glycosylphosphatidylinositol	27.6	16.58
PKMR155	Kashmiri	*OR2A12*	Chr7:143792562G>A	NM_001004135.1	c.362G>A	p.(Arg121Gln)	Missense	Olfactory receptor	24.1	16.57
PKMR159	Punjabi	*AACS*	Chr12:125612785A>G	NM_023928.3	c.1388A>G;	p.(Asn463Ser);	Missense	Acetoacetyl-Co synthetase	21.2	82.95
			Chr12:125621351C>T		c.1822C>T	p.(Arg608Cys)	Missense		34.0	198.1
PKMR164	Punjabi	*GGN*	Chr19:38877823G>A	NM_152657.3	c.79C>T	p.(Arg27Cys)	Missense	Germ cell specific gene	25.9	362.3
PKMR174	Siraki	*MEGF9*	Chr9:123421769C>T	NM_001080497.2	c.686G>A	p.(Gly229Asp)	Missense	Receptor	26.8	59.54
PKMR195	Siraki	*WFDC1*	Chr16:84360517G>A	NM_021197.3	c.634G>A	p.(Gly212Arg)	Missense	Protease inhibitor	24.2	65.89
PKMR198	Punjabi	*METTL4*	Chr18:2554909G>T	NM_022840.4	c.588C>A	p.(Cys196*)	Nonsense	Methyl transferase	NA	41.19
PKMR200	Siraiki	*UBE2J2*	Chr1:1203295_96delTT	NM_194315.1	c.77_78delAA	p.(Lys26Argfs*30)	Frameshift	Ubiquitin protein ligase	NA	0
PKMR206	Siraiki	*CCDC82*	Chr11:96117539delC	NM_024725.3	c.373delG	p.(Asp125Ilefs*6)	Frameshift	Unknown	NA	0
PKMR213	Siraiki Baloch	*TMEM222*	Chr1:27657230G>A	NM_032125.2	c.214G>A	p.(Gly72Ser)	Missense	Transmembrane protein	34.0	8.237
PKMR215	Siraiki	*PUS7*	chr7:105148870_71delTG	NM_019042.3	c.89_90delCA	p.(Thr30Lysfs*20)	Frameshift	Pseudouridylate synthetase	NA	8.237
PKMR258	Punjabi	*AREL1*	Chr14:75150203G>A	NM_001039479.1	c.277C>T	p.(His93Tyr)	Missense	Ubiquitin-protein ligase	23.1	1102
PKMR298	Punjabi	*SEPT6*	ChrX:118797529T>C	NM_145799.3	c.257A>G	p.(Tyr86Cys)	Missense	Filament formation	26.4	11.41
PKMR318	Punjabi	*DUOX1*	Chr15:45444197G>T	NM_175940.2	c.3140G>T	p.(Cys1047Phe)	Missense	Oxidase	22.9	16.59
PKMR320	Punjabi	*SLC7A10*	Chr19:33700282_83dupA	NM_019849.2	c.1372_1373dupA	p.(Thr458Asnfs*71)	Frameshift	Transporter	NA	16.49
PKMR325	Punjabi	*TM2D3*	Chr15:102182749G>A	NM_078474.2	c.677C>T	p.(Thr226Met)	Missense	Cell death or proliferation	31.0	8.344
PKMR326	Pathan	*PRKAR2B*	Chr7:106797706G>T	NM_002736.2	c.1060G>T	p.(Ala354Ser)	Missense	Kinase modulator	32.0	8.247
PKMR396	Punjabi	*RGR*	Chr10:86008779G>A	NM_002921.3	c.350G>A	p.(Arg117His)	Missense	Retinal G-protein coupled receptor	26.5	107.3

Abbreviations: GPI, Glycophosphatidylinositol; NA, Not Applicable.

^a^Chromosomal position according to GRCh37/hg19.

^b^This frameshift allele of *CAPN12* has been documented in apparently healthy British–Pakistani individual in a homozygous fashion.^35^

In family PKMR159 a compound heterozygous change was detected in *AACS* and both variants have a frequency <0.0002 in the ExAC database^27^ (Table 2). *In silico* programs support a pathogenic nature of these variants, suggesting that both alleles are pathogenic and may contribute to the ID phenotype. In family PKMR72 a synonymous change (c.2232A>G, p.(Arg813Arg)) was identified in *MDGA2* in the acceptor splice site of exon 13, which is predicted to affect splicing by three splice prediction programs (NNSPLICE^30^, HSF^31^ and MaxEntScan^32^).

In eight families, variants in more than one gene were identified (Tables 3a and 3b,; Supplementary Figure S6). Four of these genes have already been implicated in a neurological disease (*TMEM67*, *FGFR1*, *FRAS1* and *EXOSC8*), but most variants affect genes that have not previously been connected to human disease phenotypes. Of the 19 co-segregating variants, 18 are missense variants and 1 is a splice site mutation. The identification of multiple variants co-segregating with the disorder suggests the possible existence of composite phenotypes (Supplementary Table S6), which might be a relatively common phenomenon in consanguineous populations.

**Table 3a:** Multiple known genes segregating in same families

*Family*	*Ethnicity*	*Genes*	*Position* ^a^	*Transcript*	*cDNA mutation*	*Protein change*	*Variation*	*Protein function*	*CADD score V1.3*	*ExAC allele frequency x10* ^− *6*^	*OMIM*
PKMR61b	Punjabi	*TMEM67*	Chr8:94827616G>A	NM_153704.5	c.2848G>A	p.(Val950Met)	Missense	Centriole migration to the apical membrane	32.0	8.516	609884
		*FGFR1*	Chr8:38271255G>A	NM_023110.2	c.2360C>T	p.(Thr787Met)	Missense	Fibroblast growth factor receptor	29.2	126.8	136350
PKMR69	Punjabi	*FRAS1*	Chr4:79443907G>A	NM_025074.6	c.10753G>A	p.(Ala3585Thr)	Missense	Extracellular matrix protein	24.4	27.21	607830
		*EXOSC8*	Chr13:37580059C>T	NM_181503.2	c.241C>T	p.(Pro81Ser)	Missense	Exosome component	28.2	173.6	606019

Abbreviations: OMIM, Online Mendelian Inheritance In Man.

^a^Chromosomal position according to GRCh37/hg19.

**Table 3b:** Multiple novel candidate genes segregating in the same family

*Family*	*Ethnicity*	*Genes*	*Position* ^a^	*Transcript*	*cDNA mutation*	*Protein change*	*Variation*	*Protein function*	*CADD score V1.3*	*ExAC allele frequency x10* ^− *6*^	*OMIM*
PKMR30	Punjabi	*DGCR8*	Chr22:20073866G>A	NM_022720.6	c.380G>A	p.(Ser127Asn)	Missense	miRNA biogenesis	22.9	8.248	609030
		*FNIP2*	Chr4:159812659G>T	NM_020840.1	c.3011G>T	p.(Trp1004Leu)	Missense	Folliculin-interacting protein	23.6	967.4	612768
		*GSTCD*	Chr4:106640368A>G	NM_001031720.3	c.578A>G	p.(Asp193Gly)	Missense	Glutathione *S*-transferase	29.8	49.49	615912
		*TOP3B*	Chr22:22318366G>T	NM_003935.4	c.1133C>A	p.(Pro378Gln)	Missense	Topoisomerase	27.6	0	603582
PKMR51b	Pathan	*CPT1B*	Chr22:51008816C>T	NM_152245.2	c.2048G>A	p.(Arg683His)	Missense	Acetyltransferase	35.0	164.2	601987
		*PHACTR1*	Chr6:13228209C>T	NM_030948.2	c.1148C>T	p.(Ser383Leu)	Missense	Phosphatase and actin regulator	23.2	0	608723
PKMR52	Pathan	*STX19*	Chr3:93733695A>G	NM_001001850.2	c.419T>C	p.(Met140Thr)	Missense	SNARE protein	24.1	82.42	—
		*TBC1D23*	Chr3:100035033T>G	NM_001199198.2	c.1687+2T>G	p.(Asp563Glyfs*33)	Aberrant splicing	Rab GTPase activator	NA	0	—
PKMR65	Punjabi	*DNAJC2*	Chr7:102956464A>G	NM_014377.1	c.1499T>C	p.(Ile500Thr)	Missense	Phosphoprotein	29.7	75.31	605502
		*LINGO1*	Chr15:77907386T>C	NM_032808.6	c.863A>G	p.(Tyr288Cys)	Missense	Cell signaling	24.6	8.285	609791
		*VAPA*	Chr18:9945020A>G	NM_003574.5	c.517A>G	p.(Arg173Gly)	Missense	Membrane protein	21.6	0	605703
PKMR120	Punjabi	*LRRC6*	Chr8:133669098A>C	NM_012472.4	c.234T>G	p.(Ile78Met)	Missense	Receptor extracellular matrix protein	24.7	185.7	614930
		*SLC45A4*	Chr8:142228631C>T	NM_001080431.2	c.955G>A	p.(Asp319Asn)	Missense	Solute carrier	24.8	375.2	—
PKMR131	Punjabi	*ESYT3*	Chr3:138191644C>T	NM_031913.3	c.2180C>T	p.(Ser727Leu)	Missense	Extended synaptotagmin	34	124.3	616692
		*CCT6B*	Chr17:33285649C>A	NM_001193529.2	c.266G>T	p.(Gly89Val)	Missense	Chaperonin-containing T-complex	31	0	610730

Abbreviations: NA, Not Applicable; OMIM, Online Mendelian Inheritance In Man.

^a^Chromosomal position according to GRCh37/hg19.

Next, to determine the functional effect of variants, especially missense, on the secondary structure, stability and interactions of novel candidate ID proteins, we performed *in silico* molecular modeling using the HOPE and Pyre2 web-based programs. On the basis of the amino-acid sequence and structural homology with proteins with resolved crystal structures, we evaluated the effect of novel missense alleles in new ID proteins (Supplementary Figure S7). The identified variants are predicted to alter the function of the encoded proteins through their effect (loss off ionic interactions, loss of external interactions, de-stabilization of the core or the provision of more rigidity) on the secondary structure (Supplementary Figure S7).

### Newly identified ARID proteins participate in essential regulatory networks

**Figure 1:**
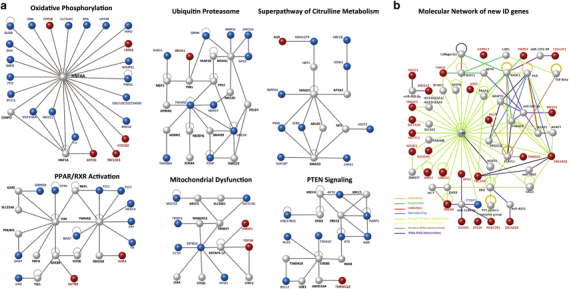
(**a**) Protein–protein interactions of novel candidates in different pathways. Novel candidate genes either directly interact with known intellectual disability (ID) genes (in blue) or through other genes (in gray) in the pathway. (**b**) Molecular functions of novel candidate genes (in red) for ID are indicated. Genes are involved in various cellular processes by activating, inhibiting and interacting with other molecules and are part of protein complexes. PowerPoint slide

Next, we applied Ingenuity Pathway Analysis^33^ to visualize the interactions between the 485 previously reported ID proteins (http://gfuncpathdb.ucdenver.edu/iddrc/iddrc/GeneQuest.php) and our novel candidate ID genes to identify the biological pathways underlying the disease process. The overall analysis, which includes physical interactions, co-expression, activation, inhibition and protein–RNA interactions, suggests that most novel ARID candidate proteins are part of already-established molecular ID gene networks (Figure 1b). By taking only physical interactions into account, we found the integration of several new ARID candidate proteins in oxidative phosphorylation, mitochondrial dysfunction, PTEN signaling and PPAR/RXR activation (*P<*0.0001; Figure 1a).

### Functional co-expression networks of ARID genes

To gain insight into the spatio-temporal expression pattern of the new candidate ARID genes, we extracted the mRNAseq data from the Allen Brain Atlas^34^ and analyzed the expression patterns in developing and adult human brain tissues (Supplementary Tables S7 and S8). All new candidate ARID genes are expressed in the human brain from a very early development stage to adulthood (Supplementary Table S8).

**Figure 2:**
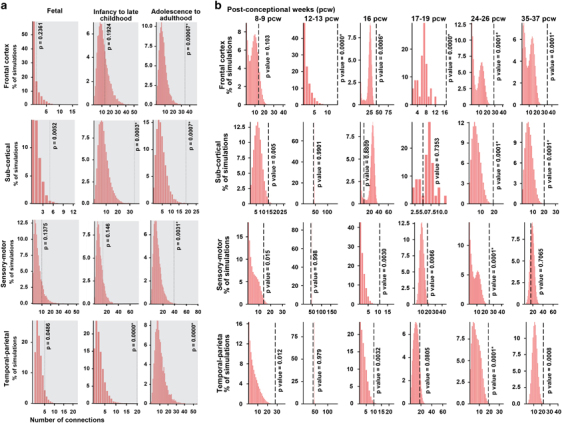
Co-expression of novel intellectual disability (ID) candidate genes was evaluated using RNA-seq data from the BrainSpan Atlas. (**a**) Gene pairs were defined as connected if the absolute value of the Pearson’s correlation coefficient is >0.8 for their expression levels in different brain region (frontal cortex, temporal–parietal, sensory–motor and sub-cortical) and different developmental stage (fetal, infancy to late childhood and adolescence to adulthood). Connections of co-expression genes at three development stages and four brain regions were plotted. Dotted lines indicate numbers of connections (edges) in networks created using target genes. Histograms represent distributions of the numbers of edges in 10 000 simulated networks using a similar number of random genes. *Represents the significance enrichment with *P* less than adjusted *P*-value. **(b)** Co-expression of novel ID candidate genes during embryonic developmental stages for the same four brain regions. PowerPoint slide

When examining the co-expression functional networks for our genes from the BrainScan database, we find that they are significantly (Bonferroni-corrected *α*⩽0.0042) co-expressed in the adult brain in all four brain regions and that they are significantly (Bonferroni-corrected *P*-value <0.0001) co-expressed in the temporal–parietal and sub-cortical regions from infancy to late childhood (Figure 2a). The phenotype observed in most of our ID families is established at birth, so to further evaluate co-expression during embryonic stages, we re-examined the normalized mRNAseq data at various post-conception weeks (p.c.w.). We found significant co-expression of ID genes in the frontal cortex region from 12 p.c.w. onwards (Figure 2b). Later in embryonic development (24–37 p.c.w.) ID genes are also co-expressed in the temporal–parietal and sub-cortical regions (Figure 2b), which persisted from infancy to late childhood (Figure 2a).

## Discussion

**Figure 3:**
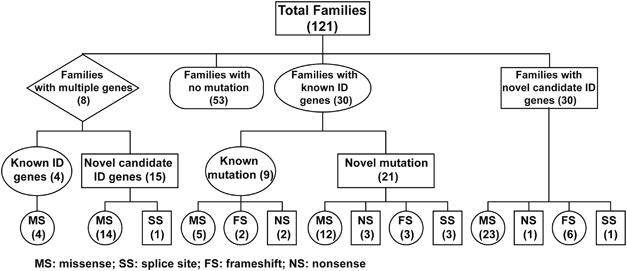
Overview of the results of genetic studies in 121 consanguineous Pakistani families segregating recessive intellectual disability (ID) phenotype. PowerPoint slide

We report the results of ES in a cohort of 121 consanguineous Pakistani families. Likely causative variants in known ID genes were identified in 30 of these families. In addition, a single likely pathogenic variant affecting genes not previously implicated in ID was identified in 30 families, and 8 families had multiple variants segregating with the ID phenotype (Figure 3). Replication of this study in independent families is warranted to add these genes to the rapidly growing list of bona fide genes implicated in ID. In fact, during the submission phase of this manuscript-independent mutations in ARID phenotypes were already reported for two genes: *DPH1* (ref. 35) and *TANGO2*.^36, 37^ Other arguments that strengthen the involvement of the candidate ID genes are the disruptive nature of missense mutations as observed by modeling of the protein structure (Supplementary Figure S7), the co-occurrence of ARID candidate genes in common regulatory pathways (Figure 1) and the co-expression of these genes in distinct regions of the human brain (Figure 2). Further credit for the involvement of the new candidate ID genes is provided by the pattern of mutations. The frequency of predicted LOF alleles (that is, nonsense, frameshift or splice site variants) in known ID genes is 13/34 (38.24%), whereas their occurrence for new candidate genes is 8/30 (26.67%), and only 1/15 (6.67%) for candidate genes in families with multiple segregating variants. These LOF variants in the new candidate genes, *METTL4*, *METTL5*, *CAPN12*, *MDGA2*, *UBE2J2*, *CCDC82*, *PUS7*, *SLC7A10* and *TBC1D23*, have the highest confidence of causality. In addition, we analyzed the exomes of 213 unrelated Pakistani control individuals for variants affecting the known ID genes and the novel candidate ID genes that we have identified in our cohort. Importantly, homozygous and compound heterozygous variants that would have passed our stringent filtering criteria were identified in eight different genes. Five of these are known ID genes (*APTX*, *ASPM*, *FLNA*, *POMT2* and *SYNE1*), and only three are new candidate ID genes, of which two were found in families with multiple segregating variants (*ESYT3* and *SLC45A4*) and one affecting a gene (*GGN*) that harbored the single segregating variant in the respective family. The relative high occurrence of LOF alleles in families with single segregating variants, as well as the paucity of predicted pathogenic variants in these genes in a control cohort provides strong support for the involvement of the candidate ID genes listed in Table 2.

Recently, ES of 3222 apparently healthy British adults of Pakistani heritage revealed 1111 rare homozygous variants in 781 genes, including a frameshift allele (c.658_659delAA) in one of the novel candidate genes *CAPN12*.^38^ No other homozygous LOF allele was observed in any novel ID candidate gene. We found the same frameshift allele (Table 2) segregating with a moderate ID phenotype (Supplementary Table S5) in two affected individuals of family PKMR64 (Supplementary Figure S4). There are at least three possible reasons to explain these observations. First, the LOF variant we identified in *CAPN12* is not causative for the moderate ID phenotype in family PKMR64, and *CAPN12* should be discarded as candidate ID gene. Second, without comprehensive assessment of mental health it is hard to rule out that the British–Pakistani individual has a mild ID phenotype similar to the phenotype in family PKMR64. Indeed, there are indications to question the health status of all individuals of the British–Pakistani cohort. For example, the reported genetic variants include homozygous LOF alleles in *AHI1*, *C12orf57* and *POMGNT1* genes, which are known to underlie the severe neurological disorders, Joubert syndrome^39^, Temtamy syndrome^40^ and muscle–eye–brain disease and other muscular dystrophy–dystroglycanopathies,^41^ respectively. Variants identified in the British–Pakistani adults also include LOF alleles in *GJB2*, *MYO3A* and *COCH* genes, which are known to cause hearing loss in humans.^42, 43, 44^ Third, there could be a protective or modifier genetic variant present in the British adult, which could render the effect of c.658_659delAA allele of *CAPN12.*^45, 46^

In the remaining 53 families, no high-confidence DNA variants was identified. This might be accounted for by several factors. First, it is possible that some causative variants have not passed our stringent filtering criteria. Second, genetic heterogeneity is likely to occur, even within some of the families we have studied. Such phenocopies would disrupt the segregation of DNA variants across all affected individuals in the studied pedigrees. Third, it is possible that some of the phenotypes with mild to moderate ID are caused by the digenic or oligogenic inheritance of rare variants or polymorphisms with low effect size. Finally, some mutations may have escaped detection by ES and may reside in non-coding regions or regions poorly covered by exonic enrichment.

### Intellectual disability and other neurodevelopmental disorders

There is a growing body of evidence that categorical neurodevelopmental disorders such as ID, autism spectrum disorders, attention deficit/hyperactivity disorders and learning disorders lack precise boundaries in their clinical definitions, epidemiology, genetics, and molecular and cellular networks.^47, 48, 49, 50, 51, 52^ Accordingly, cognitive and behavioral comorbidities such as attention deficit/hyperactivity disorders, speech delay and aggression are also frequently seen in the ID families studied here. In our study, 21.88% of the families (7 of 32) with mutations in known genes (Supplementary Tables S4 and S6) and 33.33% of the families (12 of 36) with variants in novel candidate ARID genes resulting in clinical phenotypes that include epilepsy (Supplementary Tables S5 and S6). These results are not unexpected because the prevalence of epilepsy in individuals with some degree of ID ranges from 5.5 to 35%.^53^ The prevalence of epilepsy grows with increasing severity of ID, with 15% of individuals with mild to moderate ID also exhibiting epilepsy and more than 30% of those with severe ID.^54^ This high prevalence underscores the importance of identifying largely unknown common genetic factors with a causative role in epilepsy and ARID. A striking example is provided by family PKMR82, in which a homozygous nonsense mutation (p.Arg65*) in *KCNA2* was associated with mild to moderate ID, speech delay, strabismus, walking delay and epilepsy. Recently, *de novo KCNA2* missense mutations were associated with epileptic encephalopathy, ataxia, variable ID^55, 56^ and other features reminiscent of those found in family PKMR82. These *de novo* missense mutations exert dominant effects by gain-of-function and dominant-negative mechanisms. The nonsense mutation in PKMR82 represents the first true LOF allele of *KCNA2*, which apparently has no phenotypic consequences in heterozygous mutation carriers.

### Combinatorial ID phenotypes

We observed a syndromic ID phenotype in most of the studied families (Supplementary Tables S4–S6). These syndromic ID phenotypes could be attributed to the variant of a single gene in most families, but we have also observed multiple independently segregating variants in eight families (6.6%) of our cohort (Tables 3a and 3b). This high co-occurrence of multiple potential disease-causing alleles may lead to the occurrence of composite recessive phenotypes. Both families PKMR61b and PKMR69 have segregating variants in two independent known disease genes. On the basis of the clinical presentation of previous mutations, in families PKMR61b and PKMR69 it seems that the core phenotypic features are attributable to just one of the two variants: *TMEM67* in family PKMR61b and *EXOSC8* in family PKMR69. Such inferences cannot be made for families in which segregating variants are seen in multiple novel candidate ID genes. For example, in family PKMR131 independent variants in *ESYT3* and *CCT6B* are associated with an unremarkable phenotype consisting of mild ID, speech delay, slow learning and aggressive behavior. *CCT6B* and *ESYT3* have not been connected to any human disorder so far, and therefore the variants in either or both of these genes could contribute to the phenotype.

### Functional properties of novel ARID genes

A comprehensive evaluation of the transcriptome profiles of normal human brain tissues revealed that the ID genes are significantly co-expressed in the frontal cortex region from 12 p.c.w. onwards, and in the temporal–parietal and sub-cortical regions from infancy to late childhood, implicating these regions in the pathogenesis of ID. These brain regions incorporate external and internal information and their disruptions have been associated with various disorders, such as amnesia, Alzheimer’s disease and schizophrenia,^57^ which further support the notion of shared molecular networks between various brain disorders. At later stages, we also found significant co-expression in the frontal cortex and sensory–motor regions (Figure 2a), which suggests that many of the ID genes might also have an important role in postnatal mechanisms, such as fine-tuning of synaptic connectivity, circuitry formation, and acute synaptic and other neural processes. Evidence for the involvement of postnatal mechanisms in ID is provided by studies with conditional (postnatal) knockout mice,^58, 59^ as well as studies with postnatal interventions in ID mouse models that are a basis of clinical trials for human ID disorders, such as Fragile X syndrome, Down syndrome and neurofibromatosis.^60, 61, 62^

### Common regulatory pathways involving new ARID genes

Only few of the novel ID genes seem to relate to molecular pathways and networks that have been previously implicated in dominant and X-linked forms of ID, such as pre- and post-synaptic signaling, transcription regulation and epigenetic mechanisms.^48, 51, 52^ An exception is *UBE2J2*, encoding a ubiquitin-conjugating enzyme, which represents an emergent mechanism for regulating synapse function by post-translational modification through the ubiquitin pathway at the postsynaptic membrane.^63^ Proteolysis by the ubiquitin proteosome pathway is recognized as a major molecular pathway leading to several neurodevelopmental^48^ and neurodegenerative diseases.^64^ Previously identified gene networks that are commonly disrupted in ID and other neurodevelopmental disorders are composed of genes that are highly dosage-sensitive. Thus, these pathways might represent other neurobiological processes than those that are affected by recessive mutations.

## Conclusion

Our study demonstrates the clinical utility of consanguineous populations for the elucidation of the molecular basis of very heterogeneous recessive disorders. Uncovering candidate genes for such disorders in inbred families will trigger the identification of matching mutations in other populations. In addition, a major strength of the present study is the presentation of the clinical profiles in conjunction with the reported candidate gene mutations. Therefore, the large collection of data presented in this manuscript is expected to facilitate the interpretation of DNA variants in diagnostic exome studies of patients with difficult-to-diagnose neurodevelopmental disorders. Future work using model systems may aid in unraveling and revealing the functional integration of different molecular networks in normal brain development and activity, which could add another level to neurological disorder diagnosis and more effective targeted therapy.

## Supplementary information

Supplementary Information (DOC 37 kb)

Supplementary Tables (DOC 305 kb)

Supplementary Table 3 (PDF 24 kb)

Supplementary Table 8 (XLS 814 kb)

Supplementary Figures (DOC 29328 kb)

Supplementary Table and Figure Legends (DOCX 14 kb)

## PowerPoint slides

PowerPoint slide for Fig. 1
PowerPoint slide for Fig. 2
PowerPoint slide for Fig. 3
