# Supplementary material for: Exome sequencing of Pakistani consanguineous families identifies 30 novel candidate genes for recessive intellectual disability
Source: Mol Psychiatry. 2016 Jul 26;22(11):1604–14. doi: 10.1038/mp.2016.109 (PMC5658665; doi:10.1038/mp.2016.109)
Supplement: Supplementary file 1 — Supplementary Information (DOC 37 kb) [file 41380_2017_BFmp2016109_MOESM125_ESM.doc]

**Supplementary Methods**

**Exome Sequencing of Pakistani Consanguineous Families Identifies 30 Novel Candidate Genes for Recessive Intellectual Disability**

Riazuddin S^1,2*,†^, Hussain M^1,3,4,5*^, Razzaq A^3,4,5*^, Iqbal Z^3*,$^, Shahzad M^1^, Polla DL^3,6^, Song Y^7^, van Beusekom E^3^, Khan AA^5^, Tomas-Roca L^3^, Rashid M^3,4,5^, Zahoor MY^5^, Wissink-Lindhout WM^3^, Basra MAR^5^, Ansar M^3,5,€^, Agha Z^3,8^, van Heeswijk K^3^, Rasheed F^5^, M. Van de Vorst^3^, Veltman JA^3,9^, Gilissen C^3^, Akram J^2^, Kleefstra T^3^, Assir MZ^4^, UK10K^10^, Grozeva D^11^, Carss K^12^, Raymond FL^11^, O'Connor TD^7^, Riazuddin SA^13^, Khan SN^5^, Ahmed ZM^1^, de Brouwer APM^3^, van Bokhoven H^3#,†^, Riazuddin S^2,4#,†^

Exome Sequencing (ES)

Exome enrichment and high-throughput sequencing were performed at the Radboudumc (Nijmegen), The Wellcome Trust Sanger Institute (Hinxton, as part of the UK10K study) and the University of Maryland (UOM). Exome sequencing at Radboudumc was carried out by using the SureSelect Human All Exon 50 Mb Kit (Agilent Technologies, Santa Clara, CA, USA) with coverage of ~21,000 genes and multiplex analysis on a SOLiD 4 System sequencing slide (Life Technologies, Carlsbad, CA). At the Wellcome Trust Sanger Institute, the capture was done with Agilent SureSelect Target Enrichment V5 (Agilent Technologies, Santa Clara, CA, USA) pull-down array. Subsequently, the Illumina HiSeq 2000 platform (Illumina, Inc. San Diego, CA) was used to perform the whole exome next-generation sequencing. At UOM**,** Nimblegen SeqCap EX Exome v2.0 Library (Roche Diagnostics, San Francisco, CA) was used for enrichment, followed by one hundred base pair paired end sequencing using an Illumina Hiseq2500 platform. Illumina base calling software v1.7 was employed to analyse the raw image files with default parameters. Data analysis was done using the Roche Newbler software (v.2.3) using human genome build hg19/GRCh37.

Filtering and Annotation

Seven major steps were taken to select all high-quality potentially pathogenic variants (Supplementary Figure S1): (i) inclusion of variants present in ≥4 reads and present in ≥80% of all reads (ii) exclusion of those variants that are present in unaffected controls sequenced at the same time, (iii) exclusion of variants within intergenic, intronic, and UTR regions, (iv) exclusion of variants present in dbSNP142, 1000 Genome, NHLBI Exome Variant Server (EVS) database or the Exome Aggregation Consortium (ExAC) database with a frequency ≥1%, (v) inclusion of loss-of-function variants (i.e. nonsense, frameshift, and splice site mutations) with a Phylo-P score ≥0, (v) inclusion of missense variants and in frame deletions and duplication with a Combined Annotation Dependent Depletion (CADD) score of ≥20, (vi) selected variants in genes that are expressed in the brain based on their EST profile in the Unigene database (transcripts per million - TPM ≥ 5), and (vii) inclusion of variants that segregate with the disease in in the respective pedigree as determined by using Sanger sequencing. Mutations and genes already reported to be involved in ID were identified using Online Mendelian Inheritance in Man (OMIM), NCBI Variation Viewer, “ID gene database project manged and run by University of Colorado Denver, and The Human Gene Mutation Database (HGMD).

Sanger sequencing

The exome sequencing results were confirmed by Sanger sequencing. Primers for the amplification of the exons carrying variants were designed using Primer3 (Supplementary Tables S8 and S9). PCR reactions were performed on 50ng of genomic DNA with Taq DNA polymerase (Invitrogen, Carlsbad, CA). PCR amplicons were purified with NucleoFast 96 PCR plates (Clontech Lab, Mountain View, CA), according to the manufacturers protocol. We used the ABI PRISM Big Dye Terminator Cycle Sequencing V3.1 Ready Reaction Kit and the ABI PRISM 3730 DNA Analyzer to perform sequencing (Applera Corp, Foster City, CA).

Web databases:

dbSNP142: http://www.ncbi.nlm.nih.gov/SNP/

1000 Genome: http://www.1000genomes.org/

OMIM: http://www.omim.org/

NHLBI EVS database: http://evs.gs.washington.edu/EVS/

CADD: http://cadd.gs.washington.edu/

Primer3: http://primer3.ut.ee

HOPE: http://www.cmbi.ru.nl/hope/

Phyre2: http://www.sbg.bio.ic.ac.uk/phyre2/html/page.cgi?id=index

Variation Viewer: http://www.ncbi.nlm.nih.gov/variation/view/

HGMD: http://www.hgmd.cf.ac.uk/ac/index.php

ID gene database project: http://gfuncpathdb.ucdenver.edu/iddrc/iddrc/GeneQuest.php
