# Supplementary material for: Exome sequencing of Pakistani consanguineous families identifies 30 novel candidate genes for recessive intellectual disability
Source: Mol Psychiatry. 2016 Jul 26;22(11):1604–14. doi: 10.1038/mp.2016.109 (PMC5658665; doi:10.1038/mp.2016.109)
Supplement: Supplementary file 2 — Supplementary Tables (DOC 305 kb) [file 41380_2017_BFmp2016109_MOESM126_ESM.doc]

**Supplementary Tables**

**Exome Sequencing of Pakistani Consanguineous Families Identifies 30 Novel Candidate Genes for Recessive Intellectual Disability**

Riazuddin S^1,2*,†^, Hussain M^1,3,4,5*^, Razzaq A^3,4,5*^, Iqbal Z^3*,$^, Shahzad M^1^, Polla DL^3,6^, Song Y^7^, van Beusekom E^3^, Khan AA^5^, Tomas-Roca L^3^, Rashid M^3,4,5^, Zahoor MY^5^, Wissink-Lindhout WM^3^, Basra MAR^5^, Ansar M^3,5,€^, Agha Z^3,8^, van Heeswijk K^3^, Rasheed F^5^, M. Van de Vorst^3^, Veltman JA^3,9^, Gilissen C^3^, Akram J^2^, Kleefstra T^3^, Assir MZ^4^, UK10K^10^, Grozeva D^11^, Carss K^12^, Raymond FL^11^, O'Connor TD^7^, Riazuddin SA^13^, Khan SN^5^, Ahmed ZM^1^, de Brouwer APM^3^, van Bokhoven H^3#,†^, Riazuddin S^2,4#,†^

**Supplementary Table S1: Distribution of various ethnicities in the enrolled families**

| **#** | **Ethnicity** | **% of total** | **Origin** |
| --- | --- | --- | --- |
| 1 | Punjabi | 68.6 | Primarily a clan of Indo-Aryan, 45% genetic markers resemble west and north Asian region; Main caste are Rajput/Jutt |
| 2 | Pathan** | 9 | Heterogeneous population; some tribes are associated with Greek ancestry/ Semitic Israeli ancestry and some are decent of ancient Aryan tribes.  Generally they have fair skin, light eyes and light hair ranging from blonde, red and light brown |
| 3 | Siraiki | 10.8 | An amalgamation of the Punjabi and Pathan people with some cultural adaptations (due to proximity) to Sindh and Baluchistan |
| 4 | Urdu Speaking | 7.8 | Migrant from India into Pakistan at independence in 1947 from Rajasthan, Bombay, Bihar, Delhi, Hyderabad, Baroda and Rajputana |
| 5 | Others*  (Sindhi 1.6)  (Afghan, Baloch, Kashmiri 2.2)** | 3.8 | Original inhabitants of ancient Sindh, originating from a socio-ethnic group of Dravidians and Aryans  Same as # 2 |

*Less than five families are grouped as others

**One group formed with people from same ancestry

**Supplementary Table S2: Summary of pedigrees for the 121 families enrolled in this study**

| **Sr.No.** | **Category** | **Families ascertained** | **Affected individuals** | | | **Consanguinity** | **Number of sibships** | | |
| --- | --- | --- | --- | --- | --- | --- | --- | --- | --- |
|  |  |  | **1-2** | **3** | **≥4** |  | **1** | **2** | **≥3** |
|  |  |  |  |  |  |  |  |  |  |
| 1 | XLID/ARID | 11 | 4 | 4 | 3 | 10 | 4 | 4 | 3 |
| 2 | ARID | 110 | 22 | 31 | 57 | 101 | 43 | 35 | 32 |
| 3 | TOTAL | 121 | 26 | 35 | 60 | 111 | 47 | 39 | 35 |

XLID: X-linked intellectual disability; ARID: autosomal recessive intellectual disability.

**Supplementary Table S3:** Analysis of known and novel ID candidate genes in Pakistani families with non-ID phenotype. (Please see the attached excel file for Table S3)

**Supplementary Table S4: Phenotype comparison of variants in the present study with reported mutations in ID genes**

| **Family** | **Gene** | **Phenotype** | **Reported Phenotype** |
| --- | --- | --- | --- |
| PKMR08 | *GNE* | Non syndromic. | Ascending muscle weakness, gait abnormalities |
| PKMR29 | *POMT2* | Moderate to severe ID with speech delay , hyperdontia, moderate hypotonia and Scoliosis | Muscular dystrophy-dystroglycanopathy (congenital with brain and eye anomalies), type A, 2, type B, 2, and type C, 2 |
| PKMR36 | *APTX* | V:14: Moderate hypotonia, autism, speech delay, small and squint eyes. V:13: mild ID, dwarf, elongated hands, flat bridge of nose. VI:1: ID, epilepsy | Truncating mutations resulted in a severe phenotype with childhood onset, whereas missense mutations resulted in a mild phenotype with relatively late age at onset |
| PKMR42 | *VPS13B* | Severe ID, speech delay, moderate hypotonia, microcephaly, ADHD, aggressive, hypotelorism | Cohen syndrome , developmental delay of varying degree, early-onset myopia, joint laxity, and facial dysmorphism, microcephaly, and neutropenia |
| PKMR51a | *TSHR* | Severe intellectually disability, birth hypoxia and delayed developmental milestones with no sitting or standing, no speech and hearing. isolated person | Hypothyroidism, congenital, nongoitrous |
| PKMR61a | *SCN1A* | Mild to moderate ID, physically weak in one sibship, congenital deafness, familial hemiplegic migraine, febrile seizures | Dravet syndrome, epilepsy, generalized, with febrile seizures plus, type 2 |
| PKMR79 | *AP4M1* | Severe ID with vertebral defect | Autosomal recessive spastic paraplegia |
| PKMR82 | *KCNA2* | Mild to moderate ID, speech delay, walking delay, epilepsy in v:3 and v:6, v:6 has strabismus | Epileptic encephalopathy, early infantile, |
| PKMR85 | *MED23* | No other clinical manifestation | Autosomal recessive mental retardation |
| PKMR86 | *FRAS1* | Moderate to severe ID, non-inflammatory muscular dystrophy, motor system problem | Fraser syndrome, FRAS1 mutations have more frequent skull ossification defects and a low insertion of the umbilical cord |
| PKMR87 | *MAN2B1* | limb defects, mild ID, speech delay | Mannosidosis, alpha-, types I and II, severe growth failure with hypotonia, psychomotor retardation, and hepatosplenomegaly |
| PKMR97 | *MFSD2A* | Moderate to severe ID, non-talkative | Microcephaly 15, primary, autosomal recessive |
| PKMR99 | *SYNE1* | Non Syndromic, abnormal dentition | Emery-Dreifuss muscular dystrophy, autosomal dominant spinocerebellar ataxia, |
| PKMR102 | *ASPM* | Microcephaly | Autosomal recessive microcephaly |
| PKMR105 | *ZNF41* | Intellectual disability | X-linked intellectual disability, language delay, ADHD |
| PKMR115 | *SRD5A3* | Not available | Mental and language retardation, hyperactive. |
| PKMR119 | *PGAP1* | Non syndromic | Autosomal recessive mental retardation |
| PKMR133 | *DOCK8* | Not available | Hyper-IgE recurrent infection syndrome, autosomal recessive. Mental retardation and developmental disability |
| PKMR151 | *TMEM67* | Severe ID, speech delay. epilepsy, motor weakness | Joubert syndrome, cerebellar vermis hypoplasia, hypotonia, developmental delay |
| PKMR152 | *WDR62* | Moderate ID, speech delay, microcephaly, limbs defect | Autosomal recessive microcephaly with or without cortical malformations |
| PKMR184 | *SPG11* | Moderate ID, weak memory, weak limbs and muscles | Autosomal spastic paraplegia, dysarthria, and peripheral neuropathy |
| PKMR188 | *ASPA* | Moderate ID, developmental delay, broad forehead | Canavan disease |
| PKMR193 | *ARL13B* | Severe ID, Speech delay, hypotonia, epilepsy in childhood, strabismus, growth retardation | Joubert syndrome, molar tooth sign, and small occipital encephalocele |
| PKMR212 | *ZFYVE26* | Mild to moderate ID, spasticity, progressive muscular weakness and ID started at 13 years of age. | Autosomal recessive spastic paraplegia, cognitive deterioration, axonal neuropathy, and white matter abnormalities |
| PKMR216 | *AP4S1* | Severe ID, Speech delay, hypotonia, epilepsy, growth retardation | Spastic paraplegia, autosomal recessive mental retardation |
| PKMR224 | *MKKS* | Moderate ID, speech delay, strabismus, retinitis pigmentosa, polydactyly, obesity. | McKusick-Kaufman syndrome, postaxial polydactyly and/or congenital heart disease |
| PKMR242 | *WDR73* | Mild to moderate ID, microcephaly, Dental anomalies, Weak limbs, defects in all affected,  Besides these featuresIV:3 has defects in vertebral column and disordered walk. In addition to above features IV:4 has weak limbs | Galloway-Mowat syndrome, severely delayed psychomotor development and cerebellar atrophy |
| PKMR264 | *FRY* | ID with hypertelorism, delayed speech | ID with hearing loss and strabismus |
| PKMR281 | *GPT2* | III:6: mild ID, speech delay, epilepsy; III:10 & III:11: slow learner & aggressive | Mental retardation, autosomal recessive 49, microcephaly, profoundly delayed development |
| PKMR321 | *FLNA* | Severe ID, speech delay, delayed childhood milestones, epilepsy, spasticity | Heterotopia, periventricular nodular heterotopia with frontometaphyseal dysplasia |

**Supplementary Table S5: Clinical phenotypes of families with variants in novel ID candidate genes**

| **Family** | **Gene** | **Clinical Phenotype** |
| --- | --- | --- |
| PKMR24 | *ZSCAN25 (ZNF498)* | ID, IV:3 convergent squint. |
| PKMR33 | *DPH1* | IV:1: Autism, aggressive, low nose bridge, active, good learner. IV:2: speech delay, autism, down slant eyes, epicanthus, motor weakness, self-talking. |
| PKMR40 | *DCTN2* | Severe ID, speech delay, ADHD, autism, epilepsy, spasticity in V:3, severe hypotonia in V:1 and V:2, can’t stand and walk, V:2 has open mouth, drooling, small incisors. |
| PKMR43 | *METTL5* | Severe ID, speech delay, ADHD, aggressive, autism in V:6, V:7, V:8 and VI:1. V:7 has abnormal dentation, large ears V:8 mild hypotonia. VI:1 has growth retardation, motor weakness. |
| PKMR45 | *TANGO2 (C22orf25)* | Severe ID, speech delay, mild hypotonia, ADHD, epilepsy, aggressive, squint in one individual. |
| PKMR64 | *CAPN12* | Moderate ID, speech delay, aggressive, large ears. |
| PKMR66 | *TBC1D8* | Not available. |
| PKMR67 | *MSS51*  *(ZMYND17)* | IV:2 and IV:3 have mild MR and aggressive while IV:3 has speech delay too. |
| PKMR72 | *MDGA2* | Non syndromic. |
| PKMR98 | *FMOD* | Microcephaly. |
| PKMR118 | *C22orf31* | Mild to moderate ID. IV:1 Speech delay, epilepsy 7yrs, shaking body, dental abnormalities and aggressive, IV:2 has speech delay, epilepsy till 3 years, IV:3 has normal speech but speech delay, epilepsy and aggressive till 7yrs. |
| PKMR142 | *SMARCA1* | Moderate ID, aggressive, speaking problem, elongated faces. |
| PKMR153 | *GPAA1* | Non syndromic. |
| PKMR155 | *OR2A12* | Moderate ID, speech delay, epilepsy, aggressive. |
| PKMR159 | *AACS* | Speech delay and epilepsy, microcephaly; VII:6 moderate ID with drooling, VII:7 severe ID with delayed CMS. |
| PKMR164 | *GGN* | V:4 Moderate ID with aggressive behavior, V:5 mild ID with speech delay, IV:1 has severe ID, speech delay, epilepsy, aggressive and V:3 has moderate ID, stutter, spasticity, aggressive. |
| PKMR174 | *MEGF9* | Moderate to severe ID, large ears. |
| PKMR195 | *WFDC1* | Moderate ID, speech delay, hypotelorism, delayed CMS, sleep problem. V:3 has small head and dysplastic large ears and V:2 has mild hypotonia with aggressive. |
| PKMR198 | *METTL4* | Mild to Severe ID, family history of misscarriage, delayed CMS, epilepsy in childhood, aggressive, dysplastic, difficulty in walking. |
| PKMR200 | *UBE2J2* | Mild to moderate ID, speech delay, delayed CMS. V:6 and V:7 have mild hypotonia and are aggressive. V:3 has cataract and epilepsy. |
| PKMR206 | *CCDC82* | Delayed CMS, moderate ID, and speech delay. V:3, V:4 and V:5 have mild hypotonia and motor weakness. V:3 and V:4 have bulbous nose; V:5 has hypotelorism and V:1 is squint. |
| PKMR213 | *TMEM222* | Moderate ID, Speech delay, Mild hypotonia, aggressive and delayed CMS. Body shivering in all females of this family. |
| PKMR215 | *PUS7* | Moderate ID with speech delay and aggressive behaviour. |
| PKMR258 | *AREL1* | Moderate ID, delayed CMS, speech delay, aggressive, ADHD and skin hypopigmentation. IV:1 has menorrhagia; IV:2 has spasticity, sleep disturbance and auto mutilation while IV:3 has auto mutilation and vertebral anomalies. |
| PKMR298 | *SEPT6* | Mild ID with large ears and microcephaly. V:4 and V:5 have mild ID, speech delay, microcephaly, large ears. V:13, V:14 and V:15 have mild ID, microcephaly, large ears and delayed CMS. |
| PKMR318 | *DUOX1* | Moderate ID, delayed CMS, slow learner, spasticity, speech delay and aggressive. IV:2 and IV:3 like to be isolated and IV:3 has short stature and growth retardation. |
| PKMR320 | *SLC7A10* | Mild to moderate ID, spasticity, aggressive behavior, delayed speech and childhood milestones. Retrognathia in V:10, V:11 and V:12. V:10 has febrile seizure, and cannot walk. V:11 has teeth protruding outwards, hypodontia, menangoseal, cannot walk and low immunity; V:12 has myopia, knees arthritis, problem in right leg, cannot walk properly and delayed CMS; V:5 epilepsy, dental carries, walking delay, seizure till age of 5. but now rare and V:7 epilepsy, violent shaking and loss of alertness during seizure. |
| PKMR325 | *TM2D3* | IV:1 Mild ID, aggressive, dental carries; IV:2 severe ID, spasticity, aggressive, speech and walking delay CMS. |
| PKMR326 | *PRKAR2B* | Moderate ID, spasticity, aggressive, no enamel on teeth. IV:5 epilepsy, speech delay, delayed CMS and does not have complete toilet training and IV:6 ADHD, Autism, speech delay. |
| PKMR396 | *RGR* | 2 affected: moderate to severe ID, aggressive, speech delay, night blindness, impaired conductance, teeth pointed inward, mild nystagmus. 1 affected: mild ID, epilepsy (from 6 months to 4 years age, not now), aggressive. All affected have night blindness, aggressive. IV:1 and IV:8 mild ID while IV:4 moderate to severe ID. IV:1 has myopia, teeth pointed inward, impaired conductance and mild deaf IV:8 epilepsy (from 6 months to 4 years age, not now), febrile seizures, violent shaking, hyperthermia induced seizures and IV:4 has speech delay, mild nystagmus, myopia, teeth pointed inward, impaired conductance with pain in ears and delayed CMS. |

**Supplementary Table S6: Clinical phenotypes of families segregating variants in multiple genes**

| **Family** | **Gene** | **Clinical Phenotype** |
| --- | --- | --- |
| PKMR30 | *DGCR8*  *FNIP2*  *GSTCD*  *TOP3B* | Severe ID, speech delay, delayed milestones, auto mutilation IV:1: mild hypotonia, aggressive, dysplastic, large ear. IV:2: Epilepsy |
| PKMR51b | *CPT1B*  *PHACTR1* | Severe ID, speech delay, severe hypotonia, ADHD, inability to hold head, stand or walk |
| PKMR52 | *STX19*  *TBC1D23* | Severe ID, speech delay, ADHD, spasticity, hypotelorism, squint, large and low set ears |
| PKMR61b | *TMEM67*  *FGFR1* | ID, physically weak, deafness and speech problem |
| PKMR65 | *DNAJC2*  *LINGO1*  *VAPA* | Mild to moderate ID |
| PKMR69 | *FRAS1*  *EXOSC8* | Mild ID, speech delay, spasticity, squint, abnormal dentition, Delayed developmental milestone |
| PKMR120 | *LRRC6*  *SLC45A4* | Mild to moderate ID, stunted growth |
| PKMR131 | *ESYT3*  *CCT6B* | Mild ID, speech delay, slow learner, aggressive, abnormal dentation |

**Supplementary Table S7: Expression Profiling of Novel ID genes in developing human brain**

| **Expression** | **No.** | **Names** |
| --- | --- | --- |
| Genes not developmentally regulated* | 21 | *AACS, C22orf31, CCDC82, CPT1B, DCTN2, DNAJC2, DPH1, ESYT3, GPAA1, LINGO1, METTL4, OR2A12, PHACTR1, STX19, TBC1D23, TBC1D8, TM2D3, TMEM222, TOP3B, VAPA, ZMYND17* |
| Developmentally up-regulated^†^ | 9 | *C22orf25, CAPN12, FMOD, LRRC6, METTL5, RGR, SLC7A10, SLC45A4, WFDC1* |
| Developmentally  down-regulated^†^ | 10 | *DGCR8, FNIP2, , GSTCD, MDGA2, MEGF9, PRKAR2B, PUS7, UBE2J2, ZNF498* |
| Genes with low expression^#^ | 6 | *C22orf31, DUOX1, ESYT3, OR2A12, STX19, ZMYND17* |
| Highly expressed genes^$^ | 10 | *DCTN2, DPH1, GPAA1, LINGO1, MEGF9, PHACTR1, PRKAR2B, TMEM222, VAPA, WFDC1* |

*On average no significant change (< 2-fold) in the RPKM (reads per kb per million reads) values.

^†^More than 2-fold change in the RPKM values.

^#^Normalized RPKM values ≤ 1.0.

^$^Normalized RPKM values ≥ 10.0.

RPKM: Reads Per Kilobase of transcript per Million mapped reads.

**Supplementary Table S8:** Spatio-temporal expression profiling of novel ID candidate genes in human brain. (Please see the attached excel file for Table S8)

**Supplementary Table S9: Primer sequences used to amplify and sequence pathogenic variants identified in known ID genes.**

| **Variant** | **Sequence** | **Product** | **Tm** |
| --- | --- | --- | --- |
| *GNE_F* | TGAAGTGATATCCCAGGCAAG | 552 | 60.1 |
| *GNE_R* | ATCCCTCCCTTGTGATCCTC |  | 60.3 |
| *FRAS1_F* | CACCTCAGATTAGTATGCTTTTCTTGT | 399 | 60.44 |
| *FRAS1_R* | AAGAGCAAAAGTTCAATGCATCA |  | 61.12 |
| *MFSD2A_F* | ATGAGACCTGGAGAGGTGCATA | 392 | 61.04 |
| *MFSD2A_R* | TTTAGGATGACTACAAACCACCTCA |  | 61.09 |
| *GPT2_F* | ATAACTGGTGGTCCTCTCCCTCT | 392 | 61.58 |
| *GPT2_R* | CTGCGCTTCCAGACTTCAACT |  | 62.02 |
| *FLNA_F* | CAGACACCCCTGCTGACCTAC | 476 | 62.06 |
| *FLNA_R* | AACCTTCCTGCCTTCTGAGAAAC |  | 62.27 |
| *WDR73_F* | CAGCTGGTAACAGGGACTGGTAG | 594 | 62.28 |
| *WDR73_R* | CTCATCCAATAGCCTGTTTTGGT |  | 61.55 |
| *FRY_F* | TTCTTCCTTTGTTATTTCGCTGA | 457 | 60.24 |
| *FRY_R* | CTGGAGAACTGGGATTTGTCTTT |  | 60.85 |
| *ASPA_F* | ATGCCTCGCTCAAGTATCTCTTT | 466 | 60.74 |
| *ASPA_R* | TGTGCTTAGATGCCTACCGAATA |  | 60.97 |
| *POMT2_F* | TCCTTCAGGTTAGGGTGCTG | 545 | 60.2 |
| *POMT2_R* | TTTGGGTGTAGAGCCTGGAG |  | 60.2 |
| *VPS13B_F* | CTCGCTACAGTGGTGCTCAG | 323 | 59.8 |
| *VPS13B_R* | GGAAATACATGCCTGGTTGG |  | 60.2 |
| *TSHR_F* | ACTCCTGTGCCAATCCATTC | 382 | 59.9 |
| *TSHR_R* | AGCTATGTGTTGGGGGTGTC |  | 59.9 |
| *SCN1A_F* | GCAACTCAGTTCATGGAATTTG | 498 | 59.6 |
| *SCN1A_R* | AGGTGGACAAGCTGCAGTG |  | 60.0 |
| *TMEM67_ex27_F* | TTCAGCAGTGTCCTGTATTATGG | 356 | 59.2 |
| *TMEM67_ex27_R* | AAGCAACTTATGCCCTCCAC |  | 59.2 |
| *FGFR1_F* | TGACCTCCAACCAGGTAAGG | 478 | 60.0 |
| *FGFR1_R* | AGGGAGAGGTGAGCTGAGTG |  | 59.6 |
| *FRAS1_F* | AATGCATGTGTTTCCCCTTC | 434 | 59.8 |
| *FRAS1_R* | TGGAGTGCAGTGGCTATTTG |  | 59.9 |
| *AP4M1_F* | CTGCCAGTCTCTCCTTCGAG | 340 | 60.3 |
| *AP4M1_R* | ATCCACTGCAGTCCTTCCTG |  | 60.3 |
| *KCNA2_F* | CCCACAGGACACCTATGACC | 362 | 60.2 |
| *KCNA2_R* | GACGCTCTTCCTCCTTGATG |  | 60.0 |
| *MED23_F* | GTTACCCAGGTTGGGTCTTG | 544 | 59.3 |
| *MED23_R* | AGGCTCATTTAAGCCCATTG |  | 59.2 |
| *MAN2B1_F* | CTGACACAGGGCTGTTGATG | 204 | 60.3 |
| *MAN2B1_R* | TGGTCACTTTCGTTGTCACC |  | 59.6 |
| *SYNE1_F* | TGAAAGATTAATGGGCAAGTCC | 387 | 60.3 |
| *SYNE1_R* | CTATTTCTCTAAAGGCGGATGC |  | 59.4 |
| *ASPM_F* | CATGTCCCACAGCGTAGAAG | 635 | 59.3 |
| *ASPM_R* | TTCGACATGCCTGGAATTATC |  | 59.9 |
| *ZNF41_F* | AATCCAGGATGACACCATCAC | 617 | 59.7 |
| *ZNF41_R* | TCCCTTTGTGTCTGCTGTTG |  | 59.9 |
| *SRD5A3_F* | TCGTCTGTTTTGCGTCTTTG | 559 | 60.0 |
| *SRD5A3_R* | AACTCTCAGCGCCTTGACC |  | 60.5 |
| *PGAP1_F* | AGACTAAGAAGCAAGCAGAAACAG | 405 | 58.6 |
| *PGAP1_R* | TCCAGACCCTCTGAACTTCC |  | 59.2 |
| *SCN4A_F* | ACTGGCAGCCATAGAACAGC | 243 | 60.4 |
| *SCN4A_R* | TGGATGGCAGACAGACAGAG |  | 60.0 |
| *TMEM67_ex2_F* | TGTGTATGTCTACCAGGATTTCAG | 242 | 58.2 |
| *TMEM67_ex2_R* | TTTCGCTGATCTCCCAAAAC |  | 60.2 |
| *WDR62_F* | GCCTTCTGACTTCTGGGTTG | 303 | 59.8 |
| *WDR62_R* | GTCTCACTGAGCCTGGAAGG |  | 60.0 |
| *SPG11_F* | AGGGTCCCTTCCTTCTTGG | 527 | 60.4 |
| *SPG11_R* | CATCACCCAGCACACTCTTG |  | 60.3 |
| *ARL13B_F* | AAGTGGCACTGTCACCCTCT | 721 | 59.8 |
| *ARL13B_R* | CCCACAAGCAATCAGAGACA |  | 59.8 |
| *ZFYVE26_F* | AAAAGCGTTTGGAAAACACC | 390 | 59.1 |
| *ZFYVE26_R* | CTCTACCAGGGCTTCTGTGC |  | 60.0 |
| *AP4S1_F* | GAAGGTCACCAATGGTTGTG | 221 | 58.8 |
| *AP4S1_R* | ACTCCAACCACAATGAAGAGAG |  | 58.3 |
| *MKKS_F* | CACATGCTGGGTCAATTTTTC | 537 | 60.4 |
| *MKKS_R* | AAAATGCTGAAGGCCACATC |  | 60.1 |
| *APTX_F* | TTGTGCAAGACACTCAACCTG | 486 | 59.9 |
| *APTX_R* | AATATGTGCCCTCAGCAAGC |  | 60.2 |
| *DOCK8_F* | GGGTGAGAACCTCCTTTTCC | 541 | 59.9 |
| *DOCK8_R* | CATGTGACAGCCAAGGTCAC |  | 60.2 |
| *FRAS1_ex69_F* | AATGCATGTGTTTCCCCTTC | 268 | 59.8 |
| *FRAS1_ex69_R* | GGAAAGCACTATTTCCCAAGC |  | 60.1 |
| *EXOSC8_F* | ATGGCCACAGTTGCCTTTAC | 485 | 60.0 |
| *EXOSC8_R* | TAGCCACTTGGGCCTCTTC |  | 60.3 |

F: Forward Primer, R: Reverse Primer

**Supplementary Table S10: Primer sequences used to amplify and sequence pathogenic variants identified in novel candidate ID genes.**

| **Variant** | **Sequence** | **Product** | **Tm** |
| --- | --- | --- | --- |
| *AREL1_F* | TTTTGTAGTAGGGACTATATGCCACA | 398 | 60.19 |
| *AREL1_R* | GGGGATTTGTTTCTCTCTGTGTT |  | 60.74 |
| *SLC7A10_F* | GTGTTCCCACCTCAGCTGCTA | 382 | 62.69 |
| *SLC7A10_R* | AGGGAGGAAAATTCCCCAGAC |  | 62.71 |
| *SEPT6_F* | CCCGATATCTGTGCTGTCTTCA | 394 | 62.46 |
| *SEPT6_R* | TTCATTTTCCCGTGTCAACAAAT |  | 62.6 |
| *RGR_F* | CATTCAGGAACACACACTCCAAG | 341 | 61.88 |
| *RGR_R* | ACACCTACCCACACTTCCCTGTA |  | 61.92 |
| *DUOX1_F* | CTAGGTTTCTTTCTCGGAAGCAG | 499 | 60.86 |
| *DUOX1_R* | CAGAGGACAGAGAGCTGGTTAGG |  | 61.8 |
| *TM2D3_F* | CAAGGCAAACAAAGTACAGATGCT | 397 | 61.84 |
| *TM2D3_R* | AGGGGAAGCCCTTTCTCAAAT |  | 62.43 |
| *PRKAR2B_F* | CATTCTAAGTCCTCAGAACCCACA | 392 | 61.73 |
| *PRKAR2B_R* | TGTTACCTTTTGTGGGGACCA |  | 62.43 |
| *ZNF498_F* | CTTCCTCTCCCCTGAAGCTG | 440 | 61.4 |
| *ZNF498_R* | TCACAATTACACCTCGTGGG |  | 59.4 |
| *DCTN2_F* | TAGGGCCCAGTCACCTAATG | 356 | 59.9 |
| *DCTN2_R* | AAGGCAGTCATGGTCTCAGG |  | 60.3 |
| *METTL5_F* | GACATAGATGAAGACGCATTGG | 437 | 59.6 |
| *METTL5_R* | TCCCACCTCAACCTTCTGAG |  | 60.2 |
| *TANGO2_F* | TCTCTATGGAGGGCCATCTG | 184 | 60.2 |
| *TANGO2_R* | AAGTCACGAGGAGCAACGTC |  | 60.5 |
| *CAPN12_F* | CAACACCCTCAGCATCTGG | 522 | 60.3 |
| *CAPN12_R* | GGGTGACCCTGGAAAAAGTC |  | 60.7 |
| *ZMYND17_F* | TGGGTCTGAGAGACTGGAATG | 433 | 60.2 |
| *ZMYND17_R* | CACCAAACTGTACTCCCTCTCC |  | 60.0 |
| *MDGA2_F* | CGGTCAGCATTAGGTCCTGT | 278 | 60.1 |
| *MDGA2_R* | CTGCACCATTATGGCTCTCA |  | 59.8 |
| *FMOD_F* | ATAAGGTGGGCAGGAAGGTC | 586 | 60.3 |
| *FMOD_R* | GCAGCTGGTTGTAGGAGAGG |  | 60.0 |
| *C22orf31_F* | TGCTTCCTGAAACCCAAAAG | 230 | 60.2 |
| *C22orf31_R* | GTCCCTCTGAGCACCTTCAG |  | 60.0 |
| *GPAA1_F* | AGCTCAGTGGGAGGGAAATC | 621 | 60.6 |
| *GPAA1_R* | TGGTGACCACATCACTGCTC |  | 60.8 |
| *OR2A12_F* | TGGGGCTCATCTACTTGGAC | 431 | 60.1 |
| *OR2A12_R* | ACAGGCCAATTTGAATACGG |  | 59.8 |
| *AACS_ex 13_F* | CATGGGCCACAATTTTTCTC | 283 | 60.3 |
| *AACS_ex 13_R* | AGAGGCTAGGGACCAGAAGC |  | 60.0 |
| *AACS_ex 17_F* | AATCCTTCGAGGAGGTGGAG | 352 | 60.6 |
| *AACS_ex 17_R* | AAAAAGGATGGCAGGGAGAG |  | 60.6 |
| *GGN_F* | ATCGGATCTTGGAGAACAGG | 379 | 59.1 |
| *GGN_R* | GAGAGGGCCGTTCTAAGGTG |  | 61.1 |
| *MEGF9_F* | GTTTAAACCAGTATGGTCCTTGAG | 289 | 58.2 |
| *MEGF9_R* | ATGTATGTAACTGCTCTGTGGTTG |  | 58.3 |
| *WFDC1_F* | ATTTGGCAGTTCCTGGTGAG | 240 | 60.1 |
| *WFDC1_R* | CCTCAACCTGGTGGTGAAAG |  | 60.5 |
| *UBE2J2_F* | GAGTCAAGCATGGCTCTGTG | 484 | 59.6 |
| *UBE2J2_R* | CCTGTGAGCTGTGACTCTGG |  | 59.6 |
| *CCDC82_F* | TTTTCCTCGCTTGATGTCTTC | 244 | 59.4 |
| *CCDC82_R* | CATTAACTCTGGCAACGGTTC |  | 59.6 |
| *TMEM222_F* | ATTGGTGTTGCTGTCGTTTG | 485 | 59.6 |
| *TMEM222_R* | AGGGGTCAAACACTGGTGAG |  | 60.0 |
| *PUS7_F* | TCCATGCTTCATCATGTCTG | 341 | 58.1 |
| *PUS7_R* | TTCTTGGAGCCTTAAAGATGG |  | 58.5 |
| *DGCR8_F* | TGCAGAGGTAATGGACGTTG | 465 | 59.7 |
| *DGCR8_R* | TCGAGCACTGCATACTCCAC |  | 60.0 |
| *GSTCD_F* | TCAGTGGACCAGGCTATGTG | 396 | 59.7 |
| *GSTCD_R* | ACATGCTCCAGAGGTGGAAG |  | 60.3 |
| *TOP3B_F* | ACCAGGTCTGGTACGTCAGC | 383 | 60.2 |
| *TOP3B_R* | GCACCTGCTGCTACCTCTTC |  | 60.2 |
| *DPH1_F* | GCGGAGTCACTTCCTAGCTG | 452 | 60.2 |
| *DPH1_R* | GTCGCAGACTTTGGTTAGGG |  | 59.7 |
| *CPT1B_F* | GGACAGGTGCTTTGGGTATG | 308 | 60.4 |
| *CPT1B_R* | ATTCTGCTTCCATGCAGGTC |  | 60.2 |
| *PHACTR1_F* | ATTCTGCTTCCATGCAGGTC | 310 | 60.5 |
| *PHACTR1_R* | GACCCAGGACCAGTCAACAC |  | 60.6 |
| *STX19_F* | CCATCTTCAGTGGTCACAAGG | 155 | 60.6 |
| *STX19_R* | TCTTTTCCAGCAACTTCAAGC |  | 59.6 |
| *TBC1D23_F* | GAGCATTCAGCCTACCAAGG | 392 | 59.8 |
| *TBC1D23_R* | TGGCTATTCCCAGAATGACTG |  | 60.1 |
| *DNAJC2_F* | AAAAGCAAAAACCGTAGTCCAG | 476 | 59.7 |
| *DNAJC2_R* | GTCTGCTTGAGGTACCACTCC |  | 58.8 |
| *LINGO1_F* | GGCACCTCAACATCAATGC | 439 | 60.1 |
| *LINGO1_R* | TTGGAGTCCAGGATGAGTGTC |  | 60.1 |
| *VAPA_F* | CATTCCCAATATCATGCAGAAG | 448 | 59.4 |
| *VAPA_R* | TCACACTTATACTGTCCTGCTCAC |  | 58.5 |
| *TBC1D8_F* | GGCAGTACTTCCTCCATTGC | 397 | 59.7 |
| *TBC1D8_R* | CAGGGAGTGGAAGTCCTCAG |  | 59.8 |
| *SCN3A_F* | CCTGGAAAAATTGCATGTAGC | 444 | 59.6 |
| *SCN3A_R* | GCAAGTAAGGTAAGGACCCAAG |  | 59.2 |
| *LRRC6_F* | GAAATGGGTCCTCCTTAGGC | 332 | 59.9 |
| *LRRC6_R* | ACTGCACCTGGCCTTCTAAC |  | 59.4 |
| *SLC45A4_F* | CCTTCCCAGACGAGGTACAG | 459 | 59.7 |
| *SLC45A4_R* | ACCCAAGCTTGCCGTAGTAG |  | 59.4 |
| *METTL4_F* | AACTGCTTTTCGGTGGTAGC | 329 | 59.4 |
| *METTL4_R* | GATCAGGGAGCTGATTTTGG |  | 59.6 |
| *SMARCA1_F* | CCTTCTTCTTCTCGCCCTTC | 270 | 60.5 |
| *SMARCA1_R* | ACCCCTGCTCCATCTAGCTC |  | 60.8 |
| *FNIP2_F* | GCATATGTGCATTGGCTTTG | 518 | 60.1 |
| *FNIP2_R* | ACAAAATCAGCAGGGAGGTG |  | 60.1 |
| *ESYT3_F* | CCTAAAGGCAAGGACAGTGC | 478 | 59.9 |
| *ESYT3_R* | ACCAACCAGAGTCCAGCATC |  | 60.1 |
| *CCT6B_F* | TGTGAAATTCTAATACCTCAGAAA | 347 | 55.0 |
| *CCT6B_R* | TTCAGGCCTCATTACCTGTC |  | 57.7 |

F: Forward Primer, R: Reverse Primer
