# Supplementary material for: Exome sequencing of Pakistani consanguineous families identifies 30 novel candidate genes for recessive intellectual disability
Source: Mol Psychiatry. 2016 Jul 26;22(11):1604–14. doi: 10.1038/mp.2016.109 (PMC5658665; doi:10.1038/mp.2016.109)
Supplement: Supplementary file 3 — Supplementary Table 3 (PDF 24 kb) [file 41380_2017_BFmp2016109_MOESM127_ESM.pdf]

Type	ID	Chromosome	Start position	End position	Reference	Variant	Gene name	Gene id	ExAC AF	ExAC AC Homozygotes	ExAC AC Hemizygotes	Abberation	Local position string	Reference Amino Acid	Mutation Amino Acid	AminoAcid position	mRNA changes	reads	variation reads	% variation	CADD
Homozygous	DNA13-06682	chr9	32986030	32986031	TA	TAA	APTX	NM_001195252	3,17E-03	0	-	Complex	IVS5+2	-	-	-1	-	16	2,1	100	-
Homozygous	DNA13-07594	chr9	32986030	32986031	TA	TAA	APTX	NM_001195252	3,17E-03	0	-	Complex	IVS5+2	-	-	-1	-	115	16,51	94	-
Homozygous	DNA13-07604	chr9	32986030	32986031	TA	TAA	APTX	NM_001195252	3,17E-03	0	-	Complex	IVS5+2	-	-	-1	-	35	2,9	100	-
Homozygous	DNA13-07606	chr9	32986030	32986031	TA	TAA	APTX	NM_001195252	3,17E-03	0	-	Complex	IVS5+2	-	-	-1	-	28	1,9	100	-
Homozygous	DNA13-07610	chr9	32986030	32986031	TA	TAA	APTX	NM_001195252	3,17E-03	0	-	Complex	IVS5+2	-	-	-1	-	18	2,8	100	-
Homozygous	48752	chr1	197102632	197102632	T	C	ASPM	NM_018136	1,49E-03	0	-	substitution	Ex6-92	Y	C	756	2267T>C	157	143	91	26,8
Compound	DNA1207389	chr1	197073672	197073672	C	T	ASPM	NM_018136	-	-	-	substitution	Ex18-642	W	*	1570	4709C>T	64	23	36	39
Compound	DNA1207389	chr1	197102632	197102632	T	C	ASPM	NM_018136	1,49E-03	0	-	substitution	Ex6-92	Y	C	756	2267T>C	19	7	37	26,8
Homozygous	DNA13-06682	chr3	138191271	138191271	C	T	ESYT3	NM_031913	4,14E-05	0	-	Substitution	Ex18+67	P	S	603	1807C>T	36	36	100	25
Homozygous	DNA1207389	chrX	153579381	153579381	G	C	FLNA	NM_001110556	5,76E-05	0	2	substitution	Ex44-27	A	G	2351	7052G>C	12	12	100	27,4
Homozygous	ZA56	chr19	38875072	38875072	G	C	GGN	NM_152657.3	7,59E-03	0	-	substitution	Ex4	Q	E	647	1939C>G	53	50	100	33
Homozygous	48772	chr14	77744827	77744827	C	T	POMT2	NM_013382	1,22E-03	0	-	substitution	Ex20-23	R	Q	686	2057C>T	26	26	100	23,3
Homozygous	DNA13-06682	chr14	77757698	77757698	T	C	POMT2	NM_013382	2,45E-04	0	-	substitution	Ex10-24	Y	C	381	1142T>C	31	31	100	23,5
Homozygous	DNA1207389	chr8	142228631	142228631	C	T	SLC45A4	NM_001080431	3,75E-04	0	-	substitution	Ex4-496	D	N	319	955C>T	36	35	97	24,8
Homozygous	DNA1207393	chr8	142228631	142228631	C	T	SLC45A4	NM_001080431	3,75E-04	0	-	substitution	Ex4-496	D	N	319	955C>T	8	6	75	24,8
Homozygous	DNA1207393	chr6	152674464	152674464	C	A	SYNE1	NM_182961	4,37E-03	5	-	substitution	Ex69+67	K	N	3729	11187C>A	126	120	95	21,6
Compound	DNA1207390	chr6	152583234	152583234	A	T	SYNE1	NM_182961	-	-	-	substitution	Ex101+68	L	Q	6302	18905A>T	46	9	20	28,3
Compound	DNA1207390	chr6	152674464	152674464	C	A	SYNE1	NM_182961	4,37E-03	5	-	substitution	Ex69+67	K	N	3729	11187C>A	125	54	43	21,6
