# Supplementary material for: Exome sequencing of Pakistani consanguineous families identifies 30 novel candidate genes for recessive intellectual disability
Source: Mol Psychiatry. 2016 Jul 26;22(11):1604–14. doi: 10.1038/mp.2016.109 (PMC5658665; doi:10.1038/mp.2016.109)
Supplement: Supplementary file 5 — Supplementary Figures (DOC 29328 kb) [file 41380_2017_BFmp2016109_MOESM129_ESM.doc]

**Supplementary Figures**

**Exome Sequencing of Pakistani Consanguineous Families Identifies 30 Novel Candidate Genes for Recessive Intellectual Disability**

Riazuddin S^1,2*,†^, Hussain M^1,3,4,5*^, Razzaq A^3,4,5*^, Iqbal Z^3*,$^, Shahzad M^1^, Polla DL^3,6^, Song Y^7^, van Beusekom E^3^, Khan AA^5^, Tomas-Roca L^3^, Rashid M^3,4,5^, Zahoor MY^5^, Wissink-Lindhout WM^3^, Basra MAR^5^, Ansar M^3,5,€^, Agha Z^3,8^, van Heeswijk K^3^, Rasheed F^5^, M. Van de Vorst^3^, Veltman JA^3,9^, Gilissen C^3^, Akram J^2^, Kleefstra T^3^, Assir MZ^4^, UK10K^10^, Grozeva D^11^, Carss K^12^, Raymond FL^11^, O'Connor TD^7^, Riazuddin SA^13^, Khan SN^5^, Ahmed ZM^1^, de Brouwer APM^3^, van Bokhoven H^3#,†^, Riazuddin S^2,4#,†^


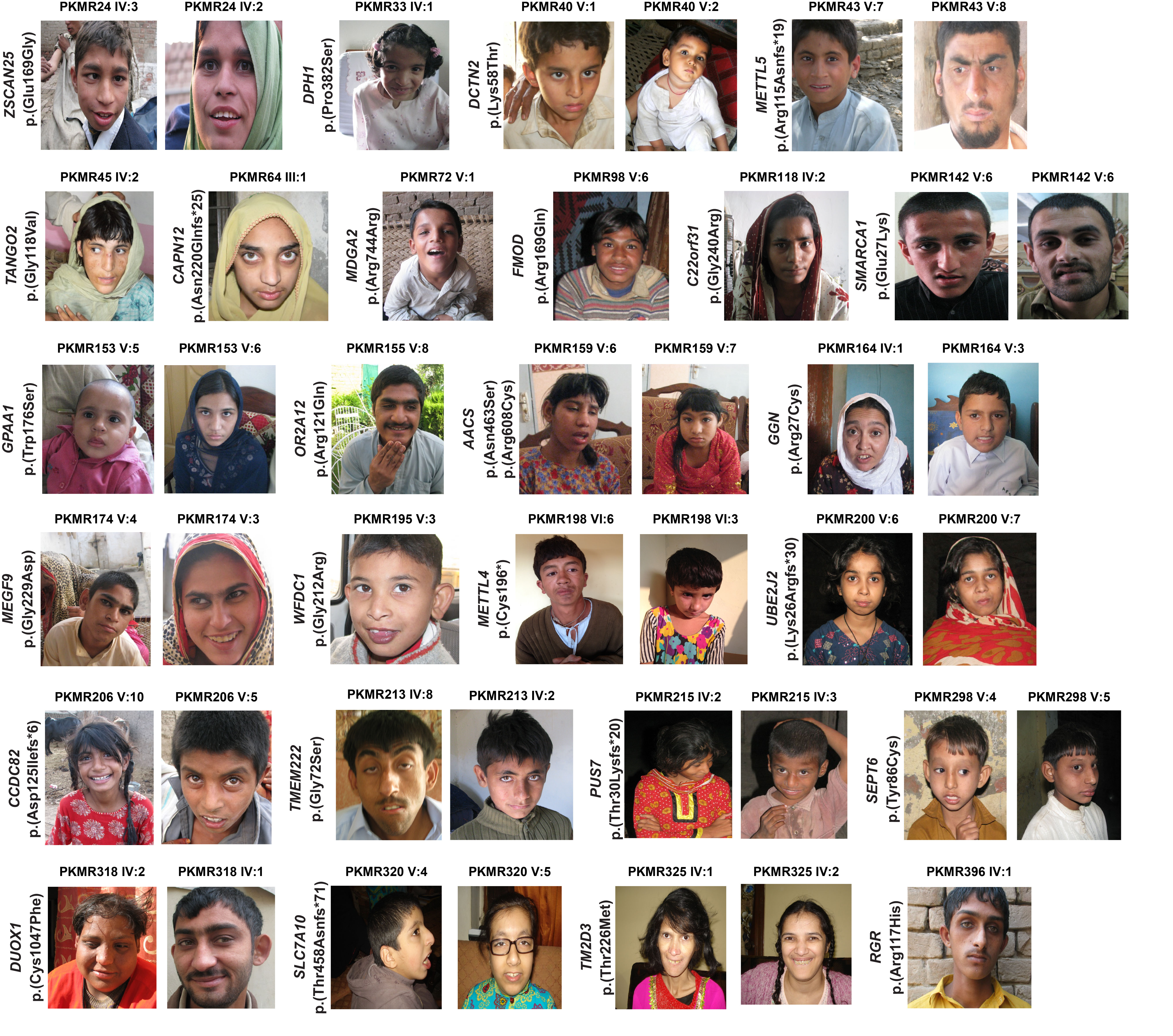


**Supplementary Figure S1: Photographs of representative affected members from 26 families. The family number and the variant identified in the novel candidate ID gene are given for each proband.**

**
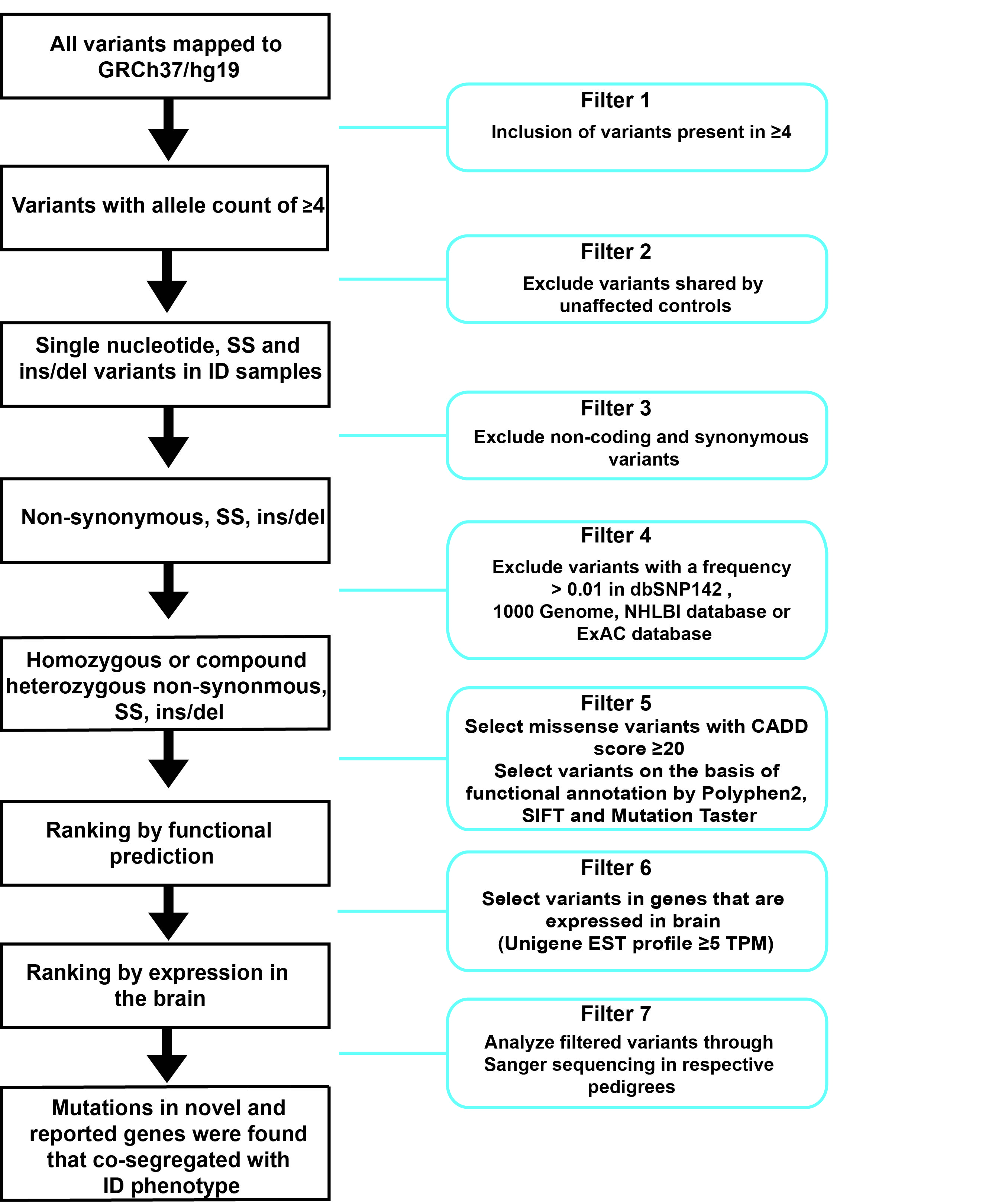
**

**Supplementary Figure S2**: A flow chart navigating through the process of filtering pathogenic variants. A seven -tier combination of different strategies is used to get plausible pathogenic variants from exome sequencing data. Frequently occurring variants with non-causative prediction are excluding and unique variants with pathogenicity clues are selected using this scheme.

**
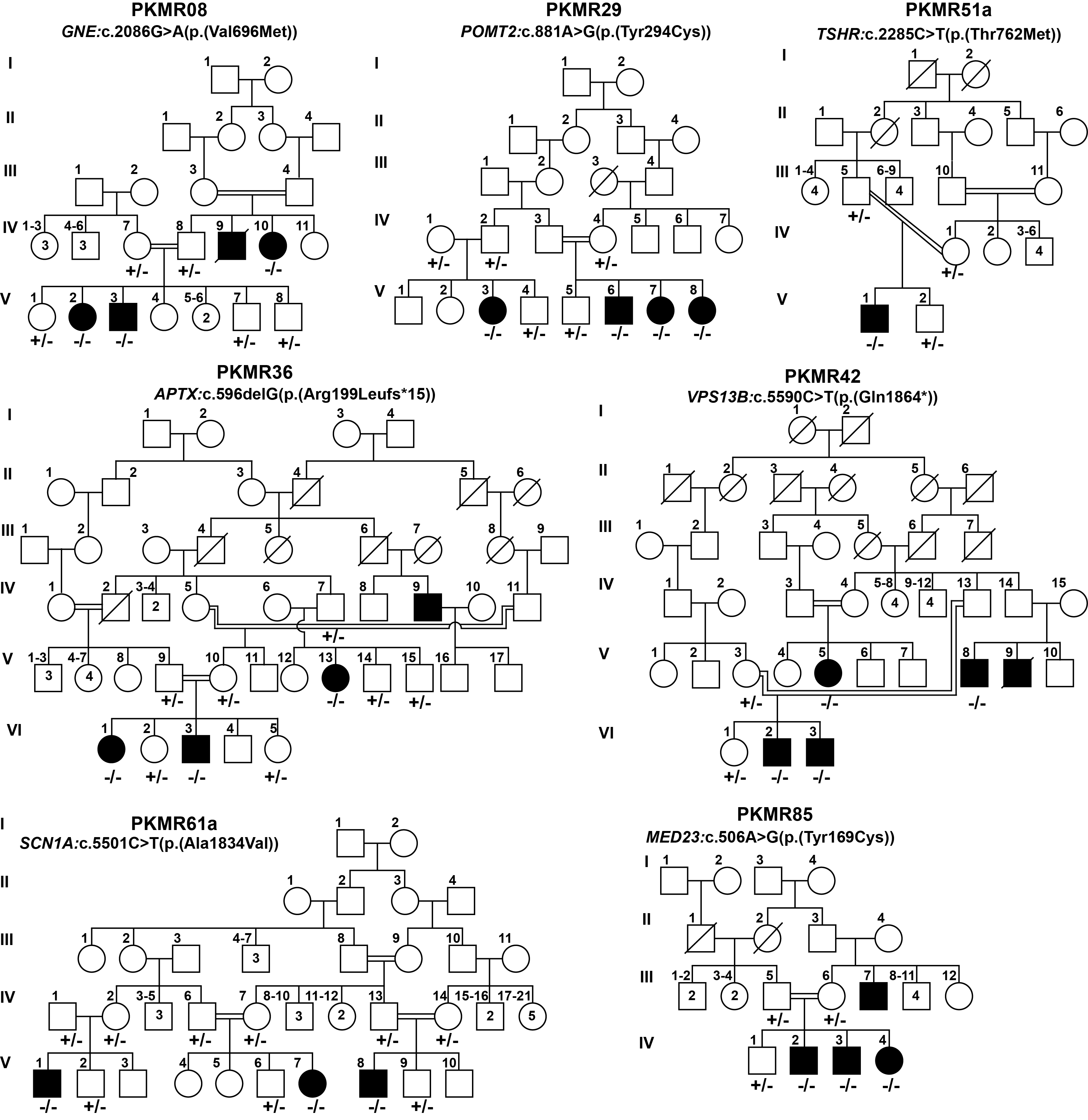

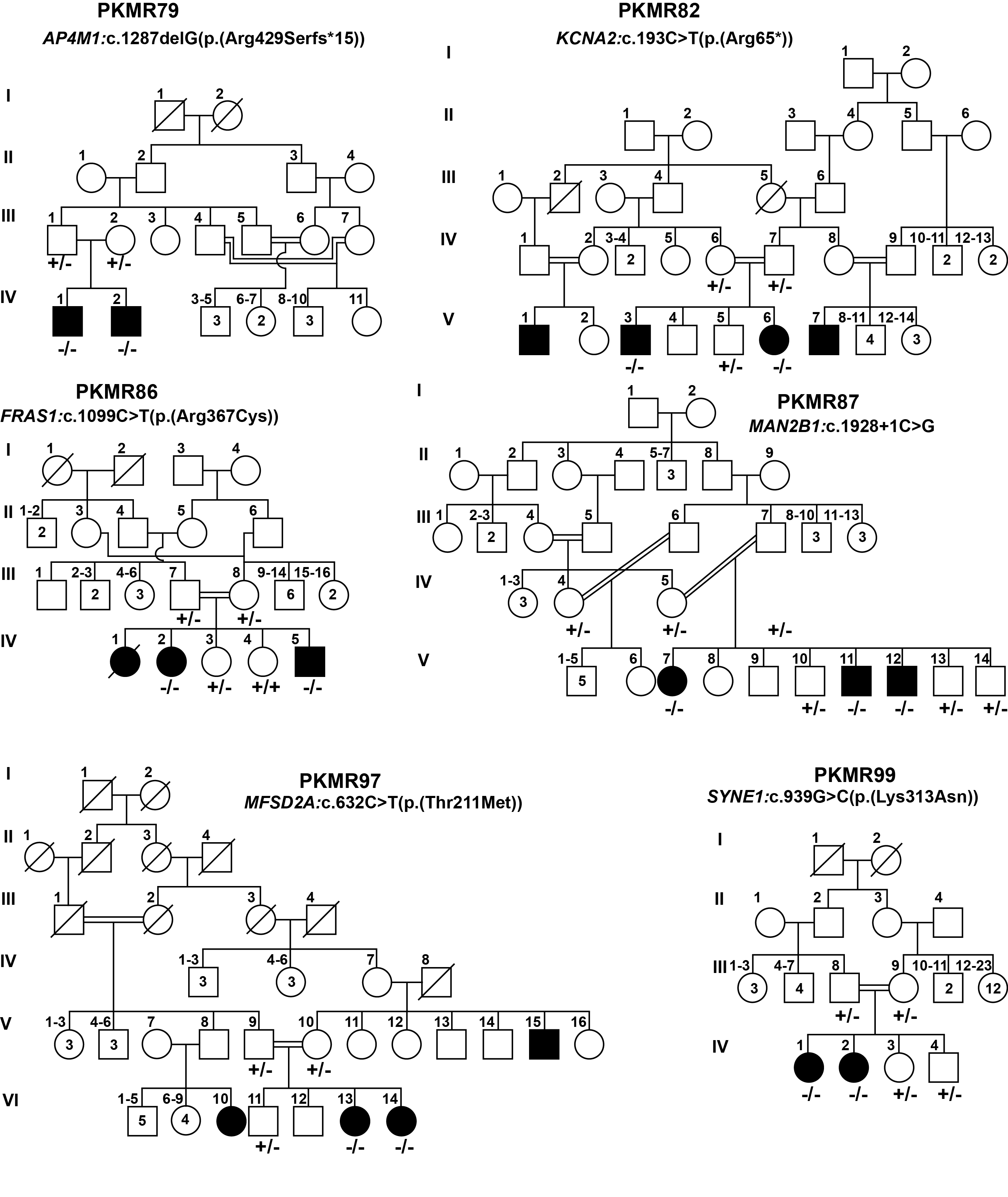

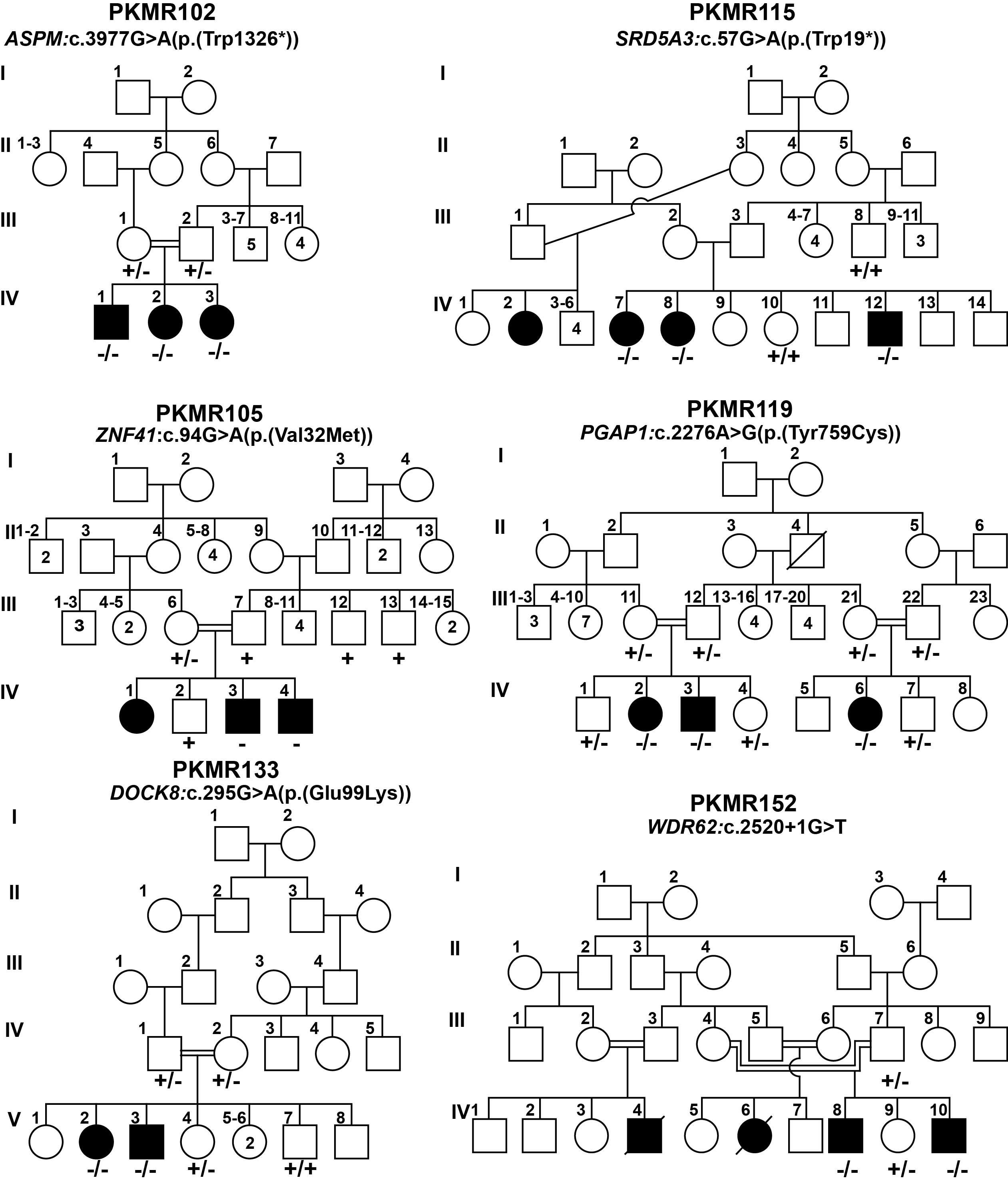

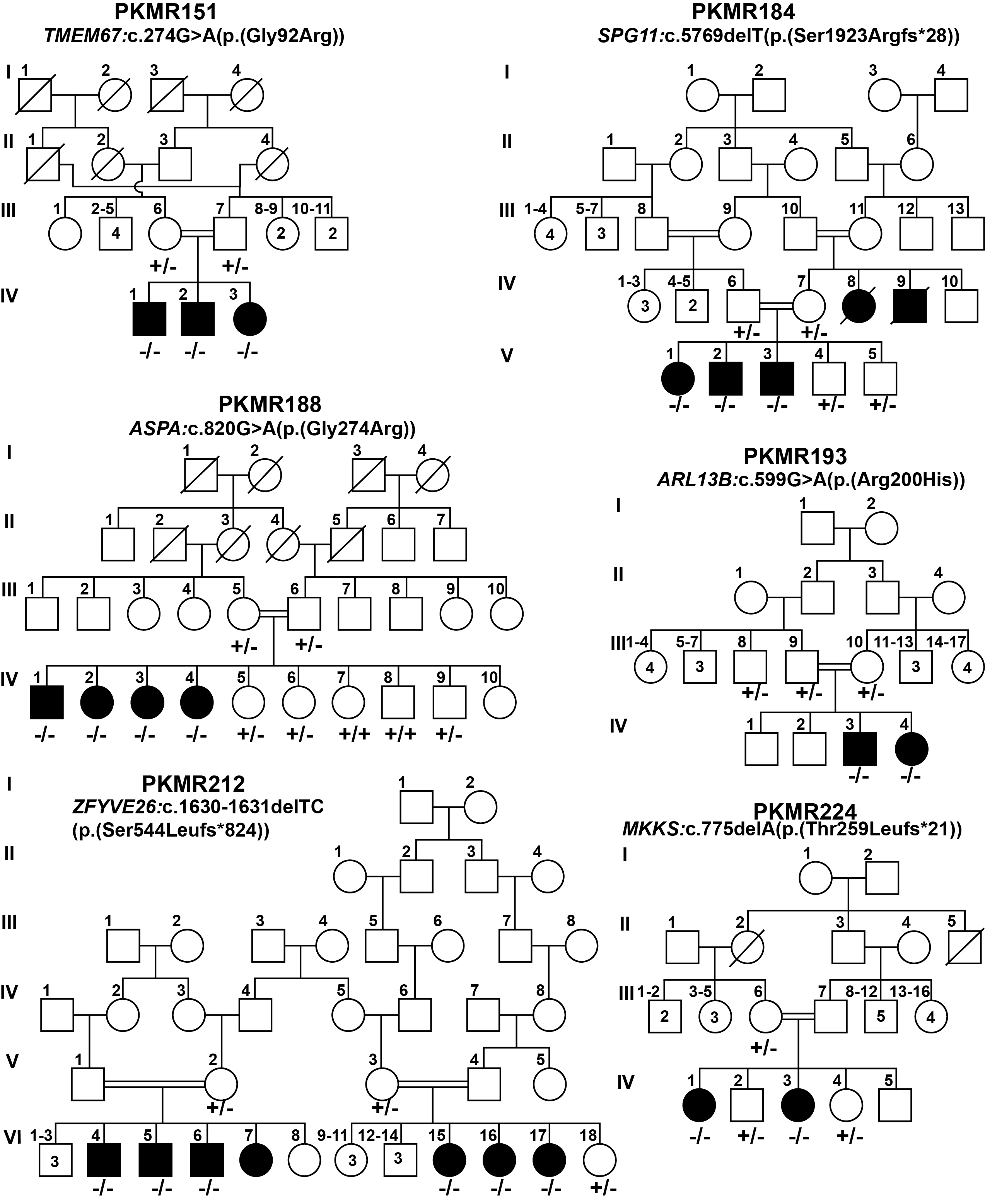

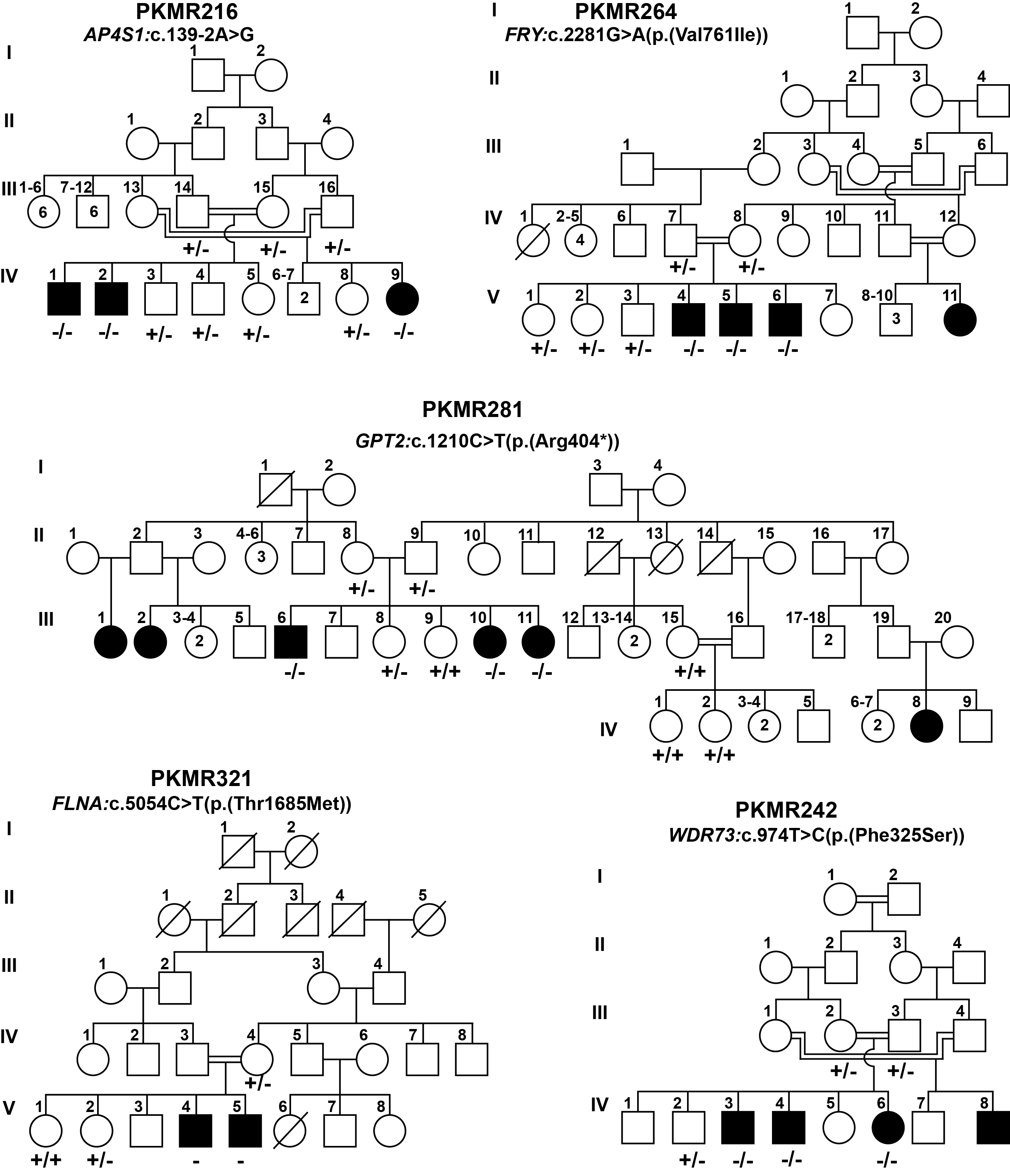
**

**Supplementary Figure S3**: Pedigrees of 30 families co-segregating recessive intellectual disability and variants in known ID genes. Filled symbols represent individuals with ID. Genotypes of the participating individuals are also shown.

**
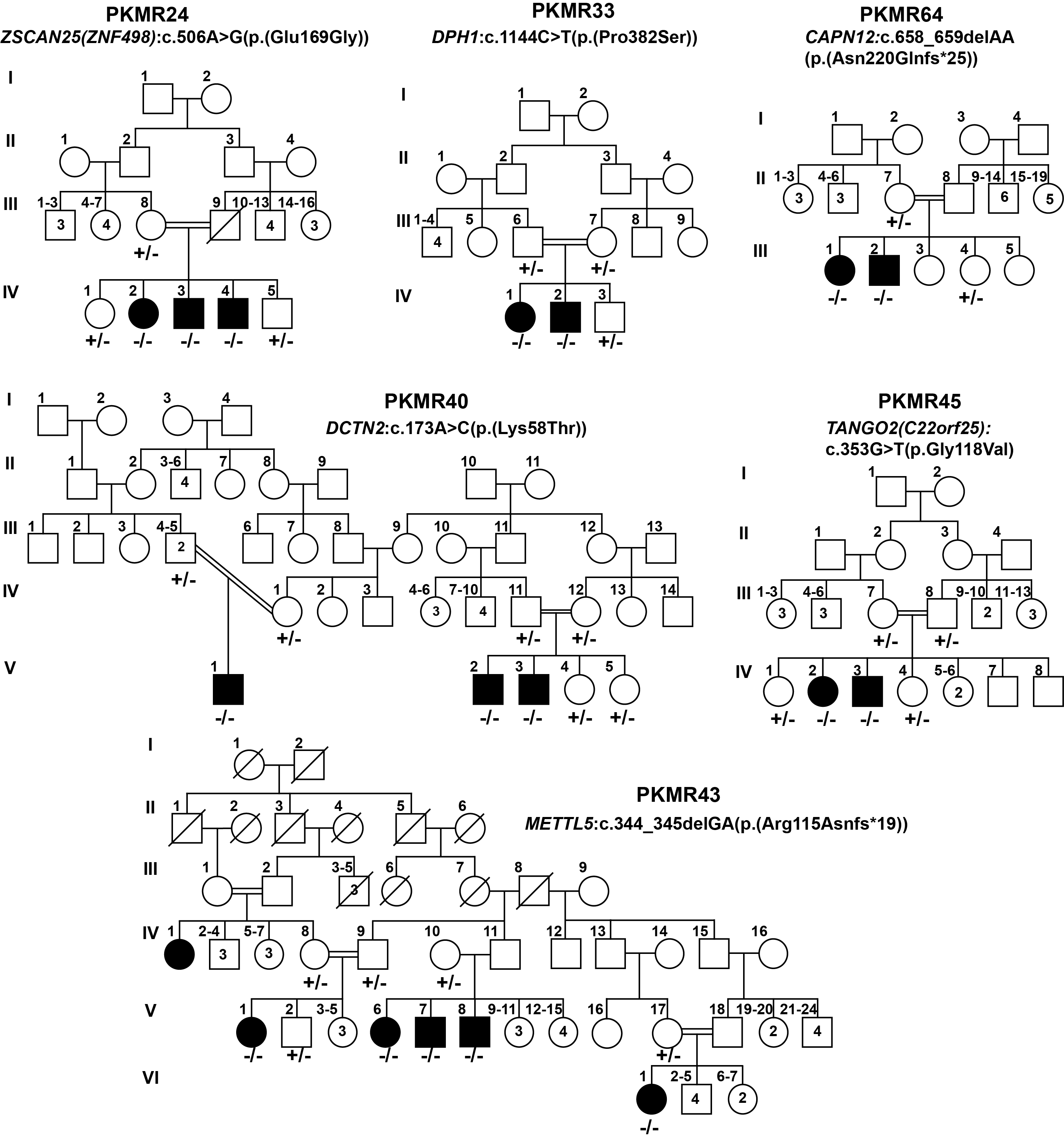
** **
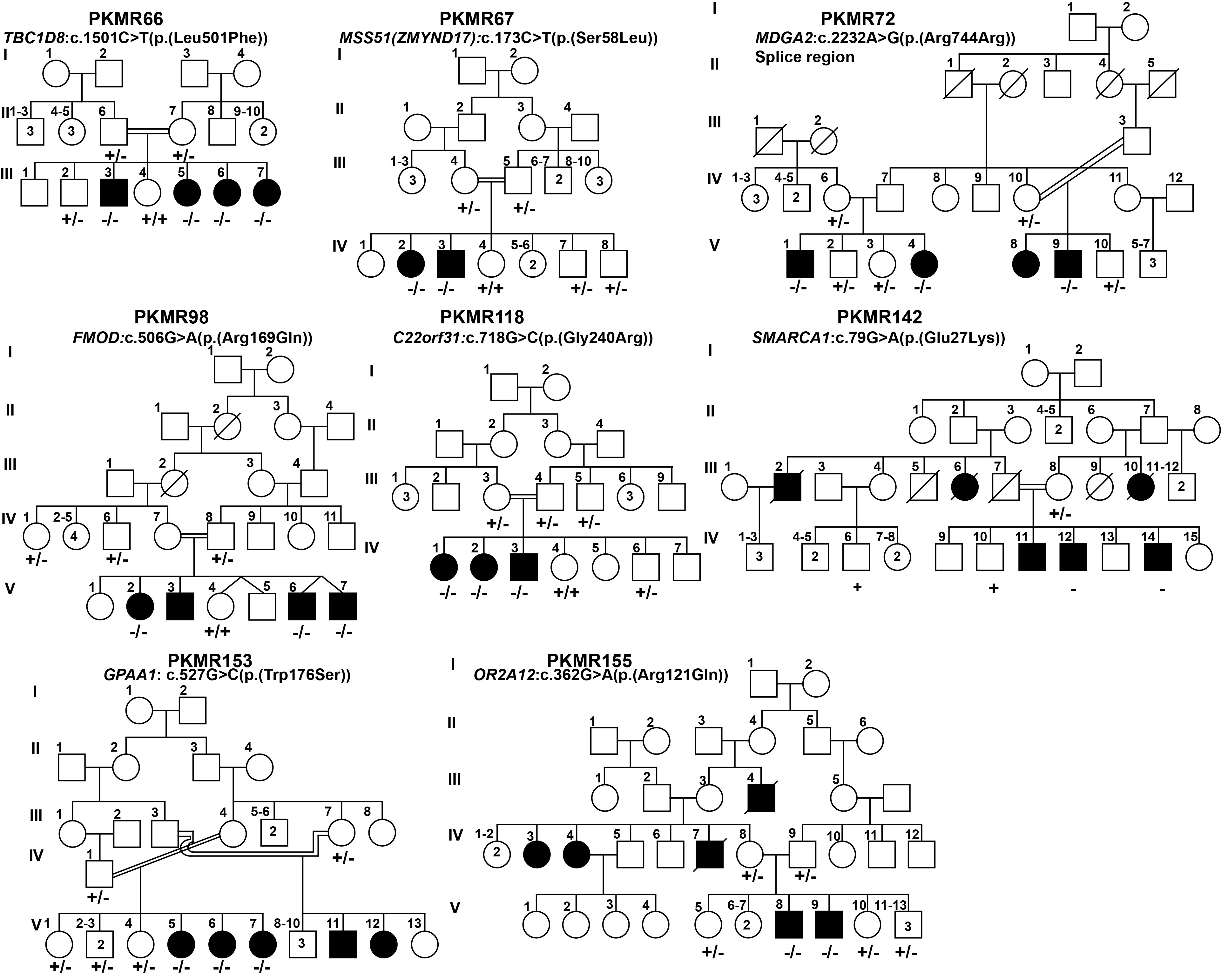
**

**
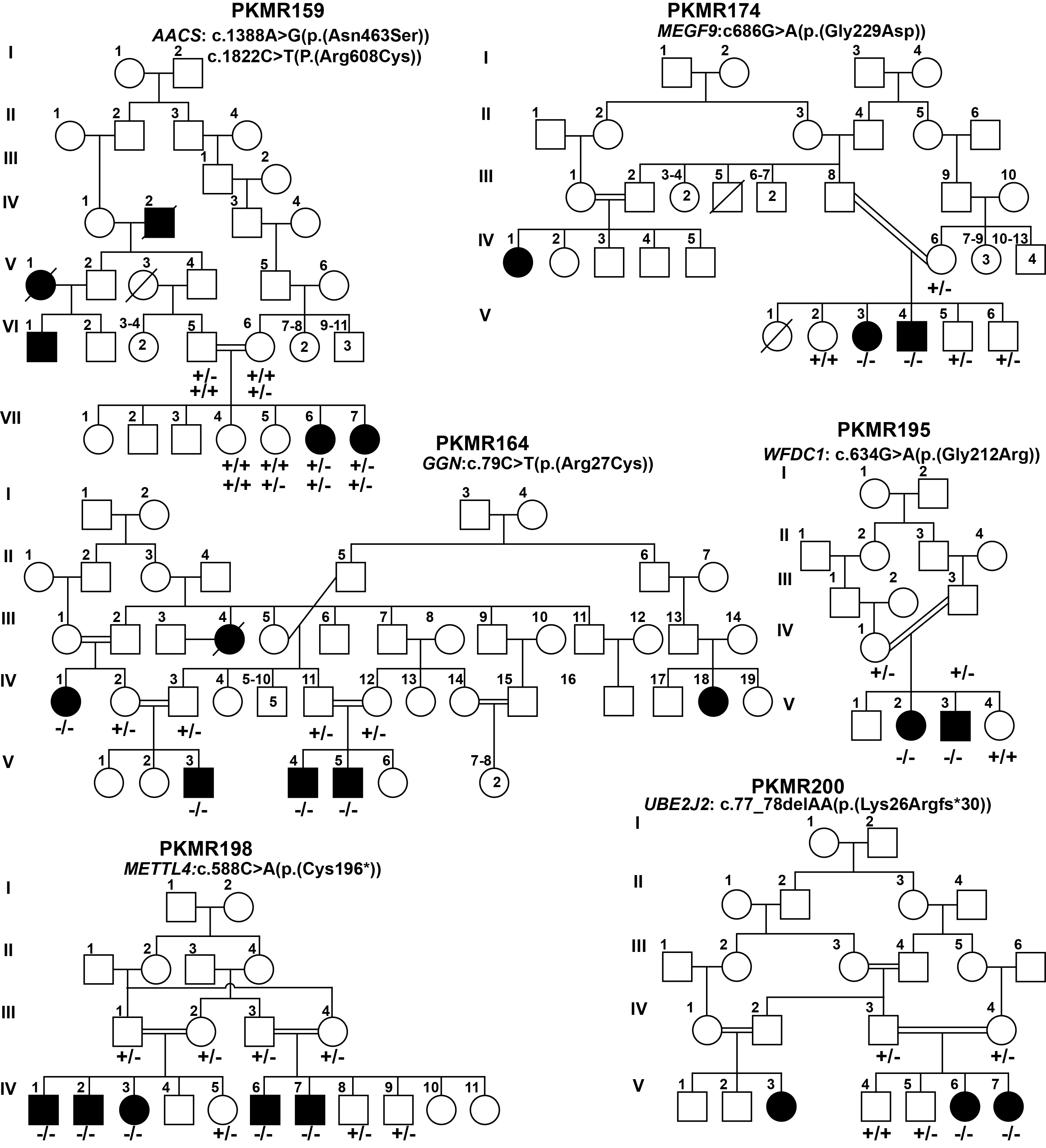

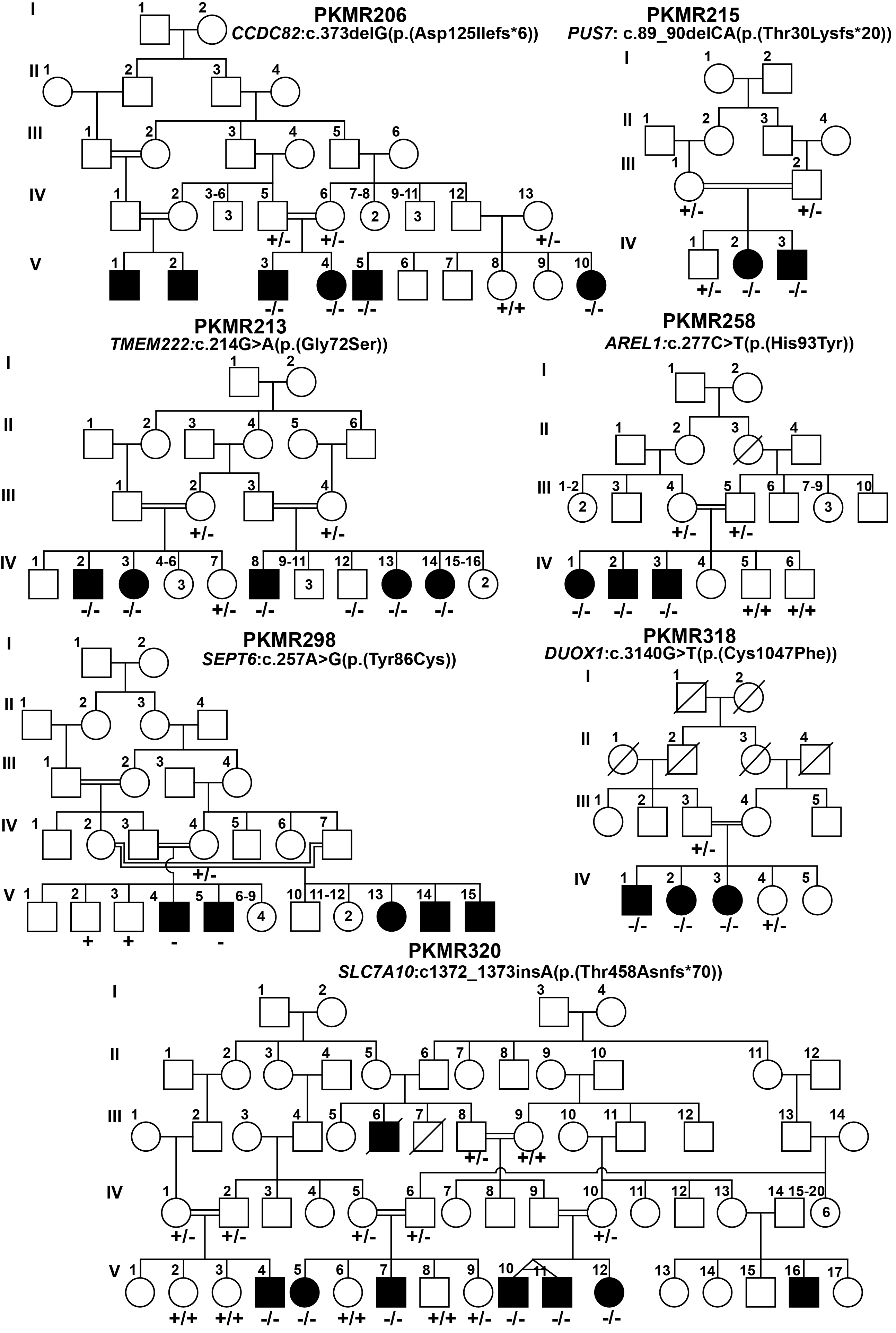

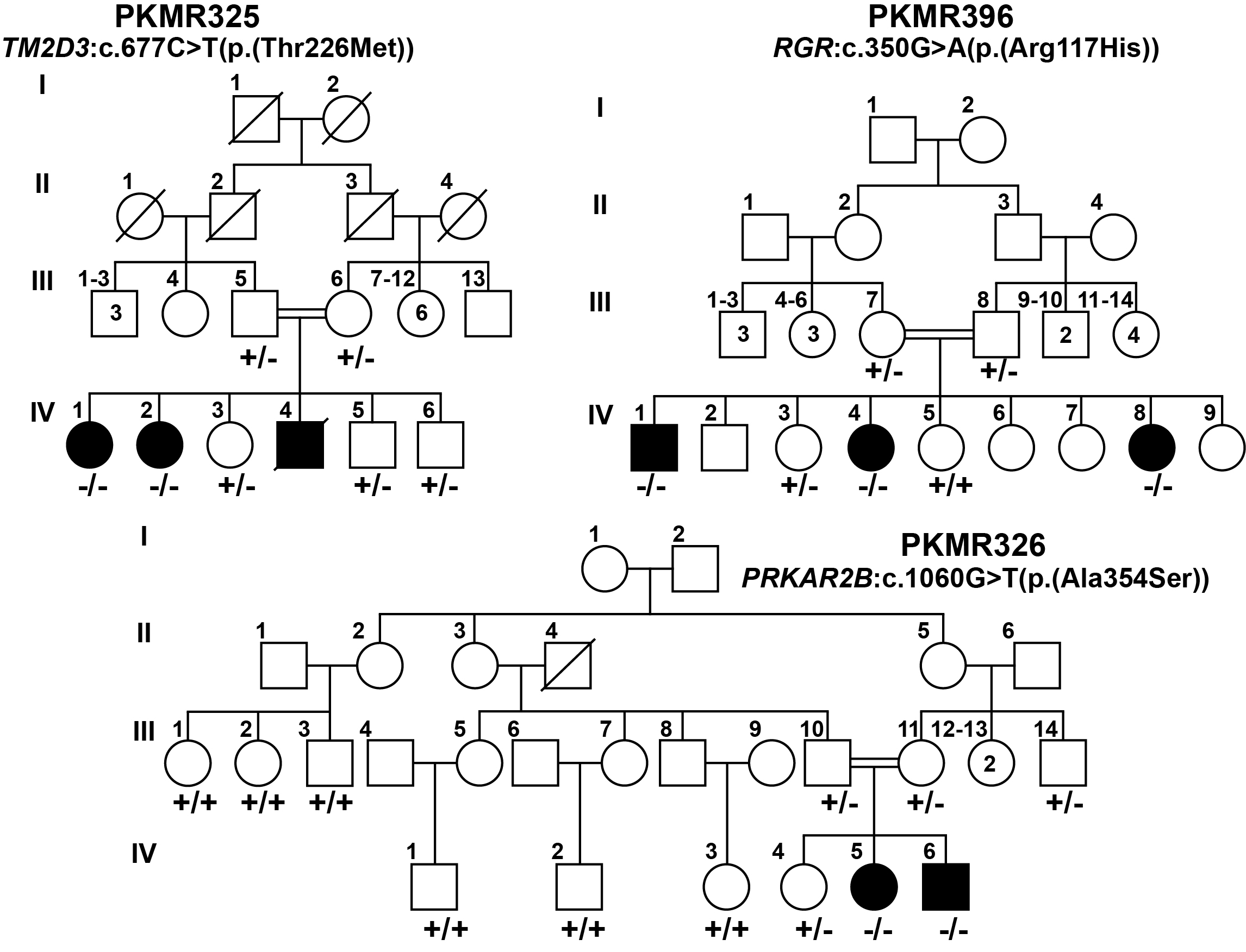
**

**Supplementary Figure S4**: Pedigrees of 30 families co-segregating recessive intellectual disability and variants in novel *ID* genes. Filled symbols represent individuals with ID. Genotypes of the participating individuals are also shown.

**
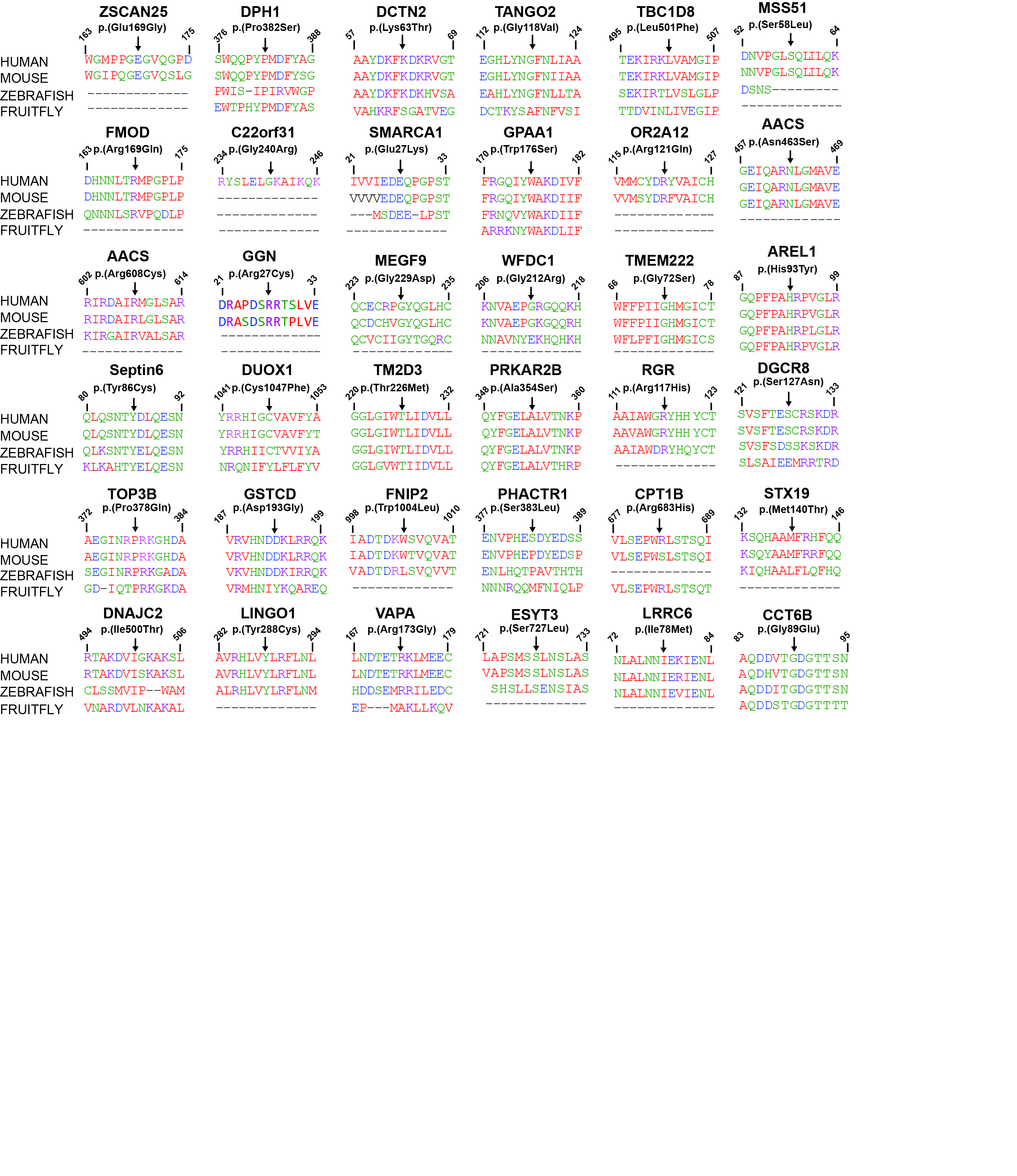
**

**Supplementary Figure S5**: Clustal W alignment of proteins family members to show conservation of amino acids mutated in Pakistani families.

**
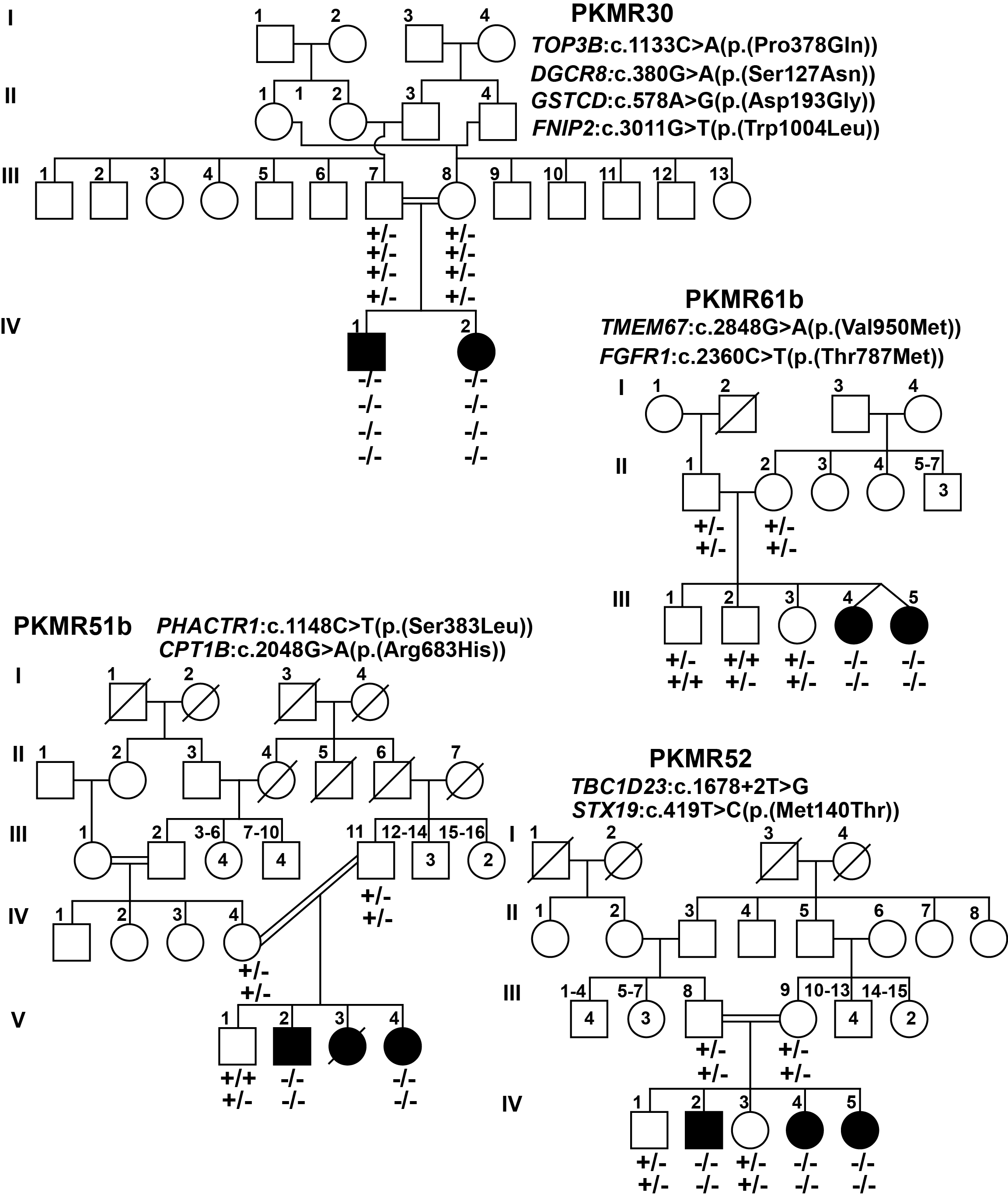

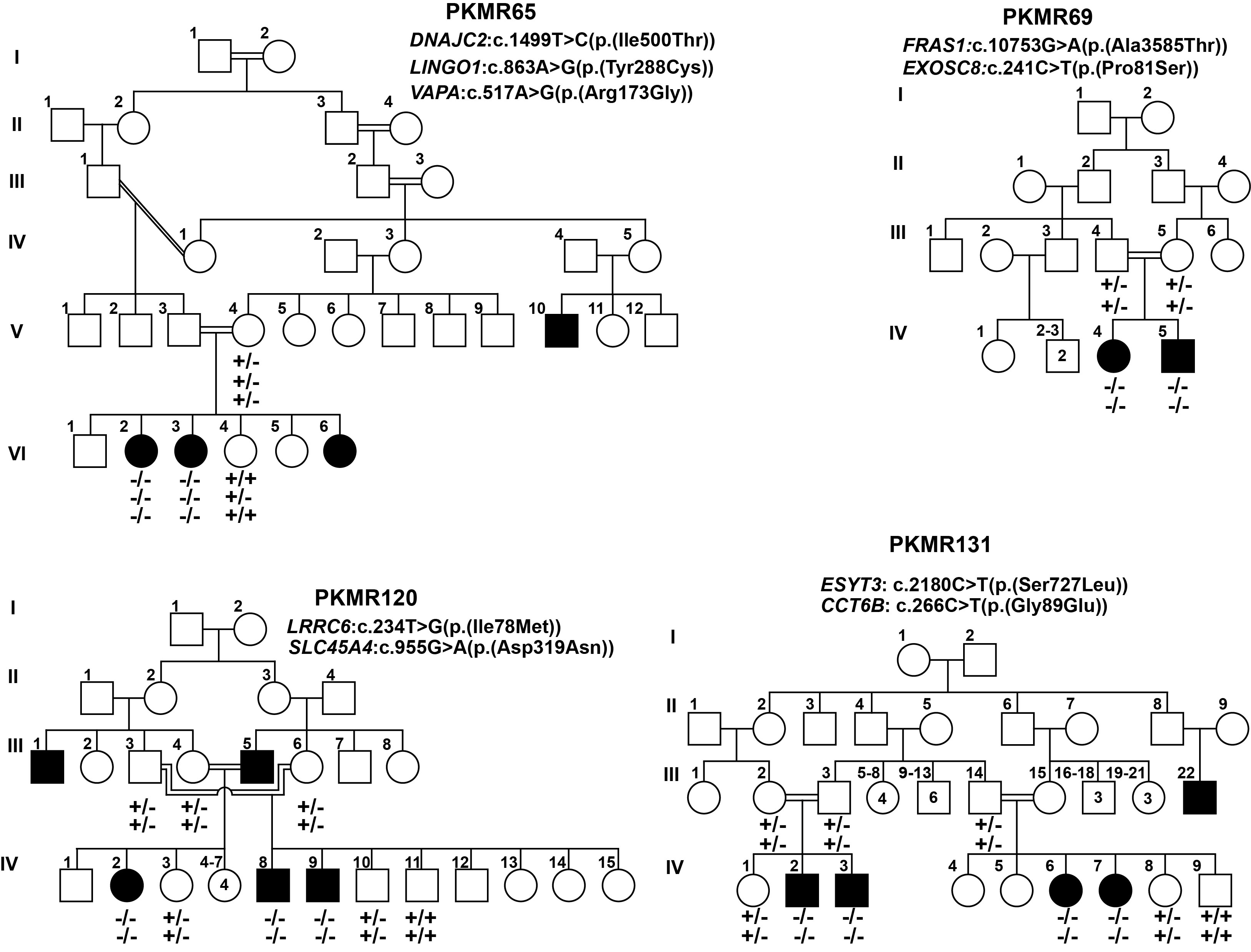
**

**Supplementary Figure S6**: Pedigrees of 8 families co-segregating recessive intellectual disability and variants in multiple candidate ID genes. Filled symbols represent individuals with ID. Genotypes of the participating individuals for each candidate gene are also shown.

**
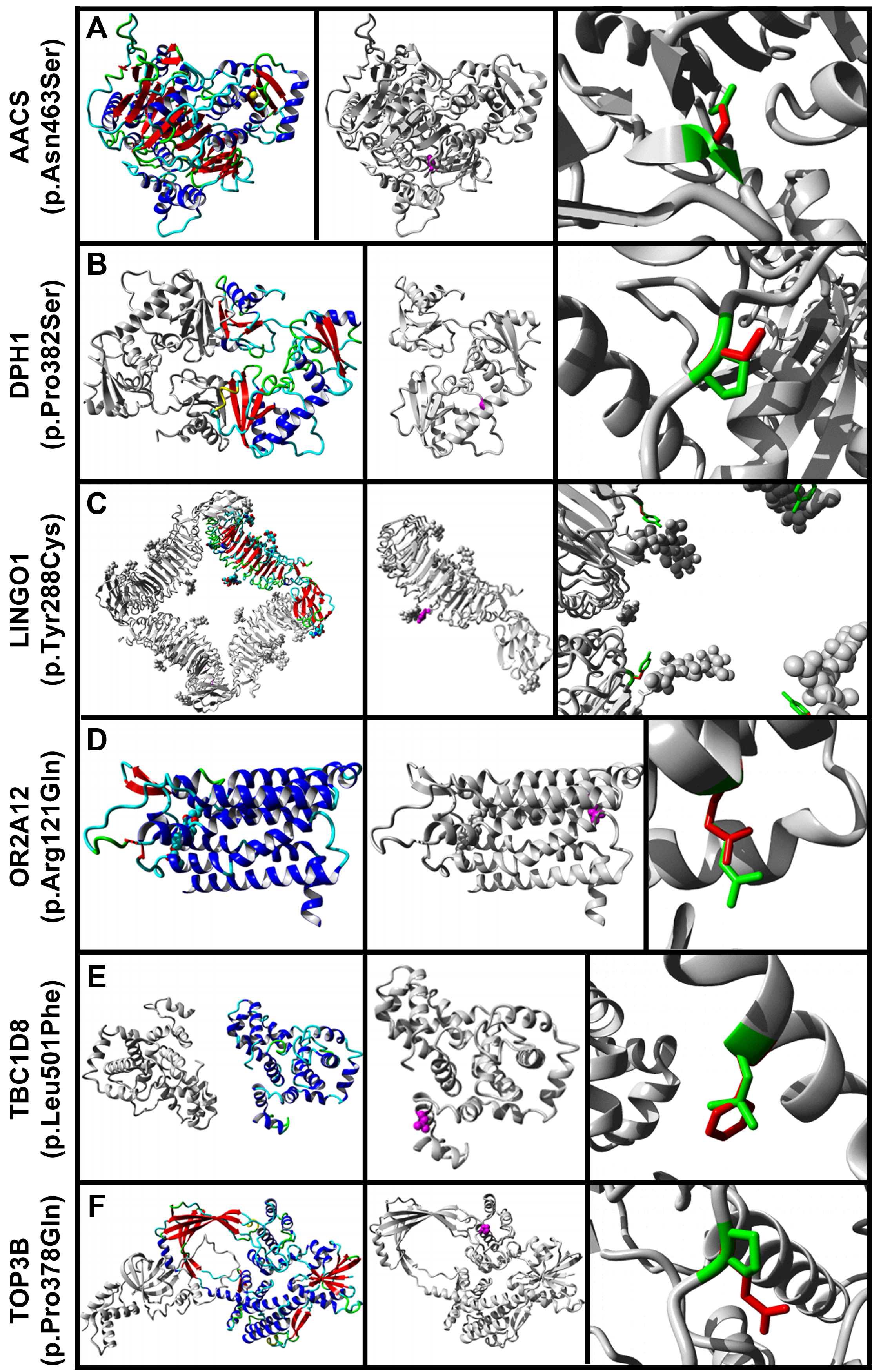
**

**Supplementary Figure S7:** Representative molecular modeling data for the novel ID genes. Right column shows the overview the respective proteins, colored by elements: α-helix=Blue, β-strand=Red, turn=green, random coils=cyan, and other molecules=grey. Middle column shows the proteins structure in grey, with the side chain of the mutated residue shown as magenta ball. Left column displays the magnified image of the wild type (green) and mutated residue (red). (**A**) Modeling for p.(Asn463Ser) change in AACS protein showed that the highly conserved wild type residue is replaced by smaller and more hydrophobic amino acid, which could result in loss of hydrogen bonds in protein core and folding might be disrupting due to creation of empty space. Amp-Dependent Synthetase/ligase domain (IPR000873) contains this mutation and domain functions in Acetoacetate-Coa Ligase activity (GO:0030729). (**B**) Molecular modelling of DPH1 suggests that backbone forming wild type p.Pro382 is replaced by smaller and more hydrophobic serine residue, which would result in loss of interactions with other molecules and conformation changes in interpro domain Diphthamide Synthesis Dph1 (IPR016435). (**C**) LINGO1 tetramerize and the p.Tyr288 residue is present leucine-rich repeat domain (IPR001611) that functions in protein binding (GO:0005515). Replacing this residue with smaller and more hydrophobic cysteine residue is predicted to result in loss of external interaction. (**D**) The p.Arg121 residue in OR2A12 protein is located in G-protein coupled receptor, rhodopsin-like domain (IPR000276) that performs G-protein coupled receptor activity (GO:0004930). Replacing the wild type arginine with smaller and neutral glutamine amino acid would create empty space in protein core and is predicted to disrupt ionic interactions. (**E**) TBC1D8 molecular modeling for mutation p.(Leu501Phe) showed that wild type residue is replaced by bigger amino acid and it would not fit inside core of protein. Mutant residue does not prefer α-helices and could disrupt interpro Rab-Gtpase-Tbc domain (IPR000195). (**F**) Molecular modeling for mutation p.(Pro378Gln) indicates that wild type residue is replaced by bigger and less hydrophobic amino acid. Conserved proline induced backbone conformation of protein would be affected by mutation. The residue is present in DNA Topoisomerase, Type 1a domain (IPR000380) and has DNA topoisomerase Type I activity (GO:0003917). Mutant residue can affect hydrophobic interactions with other molecules on protein surface.
