# Supplementary material for: Exome sequencing of Pakistani consanguineous families identifies 30 novel candidate genes for recessive intellectual disability
Source: Mol Psychiatry. 2016 Jul 26;22(11):1604–14. doi: 10.1038/mp.2016.109 (PMC5658665; doi:10.1038/mp.2016.109)
Supplement: Supplementary file 6 — Supplementary Table and Figure Legends (DOCX 14 kb) [file 41380_2017_BFmp2016109_MOESM130_ESM.docx]

**Supplementary Figures and Tables**

**Figure Legends**

**Figure S1:** Photographs of representative affected members from 26 families. The family number and the variant identified in the novel candidate ID gene are given for each proband.

**Figure S2**: A flow chart navigating through the process of filtering pathogenic variants.

**Figure S3**: Pedigrees of 30 families co-segregating recessive intellectual disability and variants in known ID genes.

**Figure S4**: Pedigrees of 30 families co-segregating recessive intellectual disability and variants in novel *ID* genes.

**Figure S5**: Clustal W alignment of proteins to show conservation of amino acids mutated in Pakistani families.

**Figure S6**: Pedigrees of 8 families co-segregating recessive intellectual disability and variants in multiple candidate ID genes.

**Figure S7**: Representative molecular modelling data for the novel ID genes.

**Table S1**: Distribution of various ethnicities in the enrolled families.

**Table S2**: Summary of pedigrees for the 121 families enrolled in this study

**Table S3**: Analysis of known and novel ID candidate genes in Pakistani families with non-ID phenotype.

**Table S4**: Phenotype comparison of variants in the present study with reported mutations in ID genes

**Table S5**: Clinical phenotypes of families with variants in novel ID candidate genes.

**Table S6**: Clinical phenotypes of families segregating variants in multiple genes.

**Table S7**: Expression profiling of novel ID genes in the developing and adult human brain.

**Table S8**: Spatio-temporal expression profiling of novel ID genes in developing and adult human brain tissues.

**Table S9**: Primer sequences used to amplify and sequence pathogenic variants identified in known ID genes.

**Table S10**: Primer sequences used to amplify and sequence pathogenic variants identified in novel candidate ID genes.
