# Supplementary figures and images for: Exome sequencing of Pakistani consanguineous families identifies 30 novel candidate genes for recessive intellectual disability
Source: Mol Psychiatry. 2016 Jul 26;22(11):1604–14. doi: 10.1038/mp.2016.109 (PMC5658665; doi:10.1038/mp.2016.109)

## Slide 1
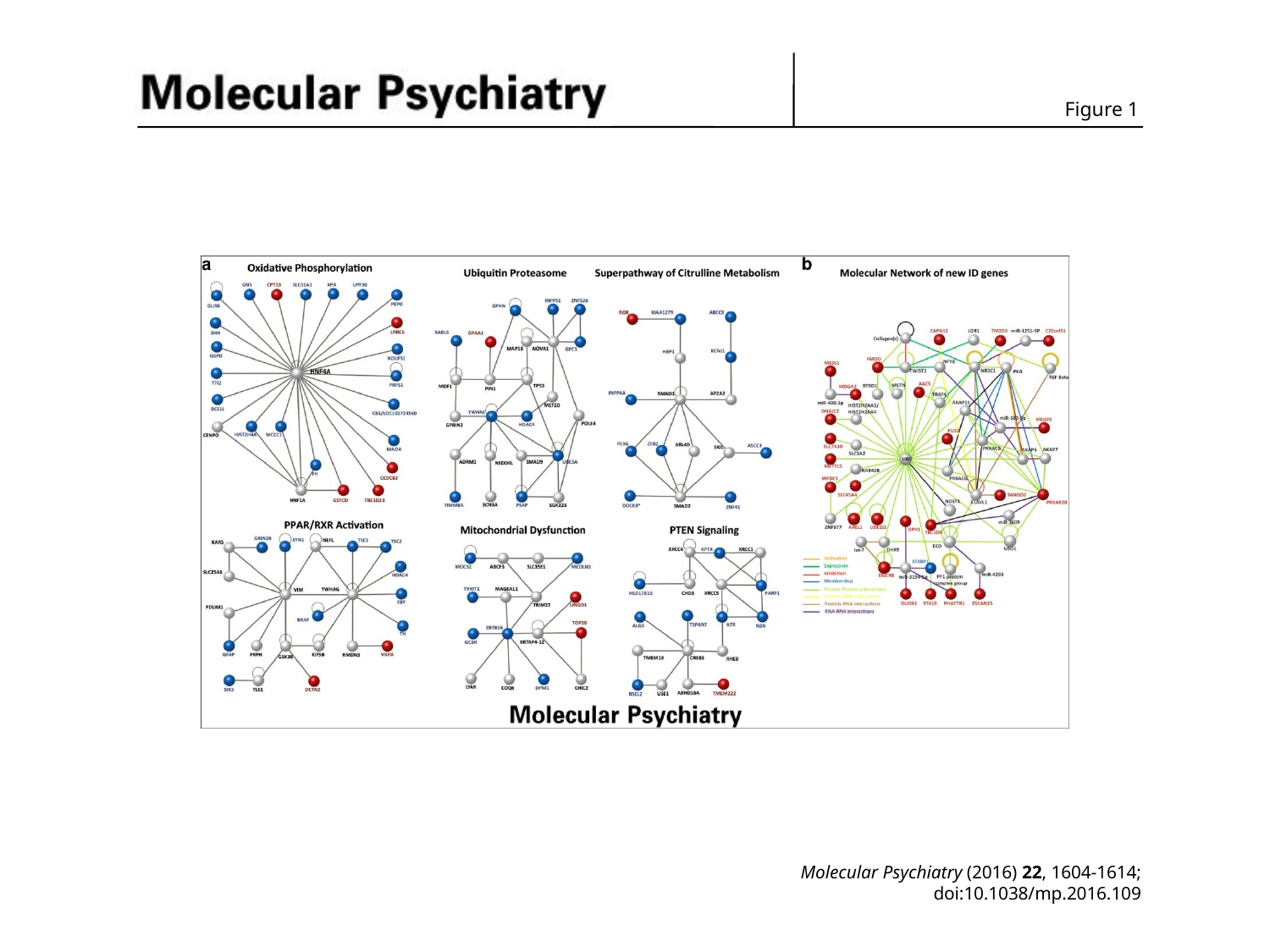

Figure 1
Molecular Psychiatry (2016) 22, 1604-1614;
doi:10.1038/mp.2016.109

Supplement: Supplementary file 7 — PowerPoint slide for Fig. 1 [file 41380_2017_BFmp2016109_MOESM122_ESM.ppt]

## Slide 1
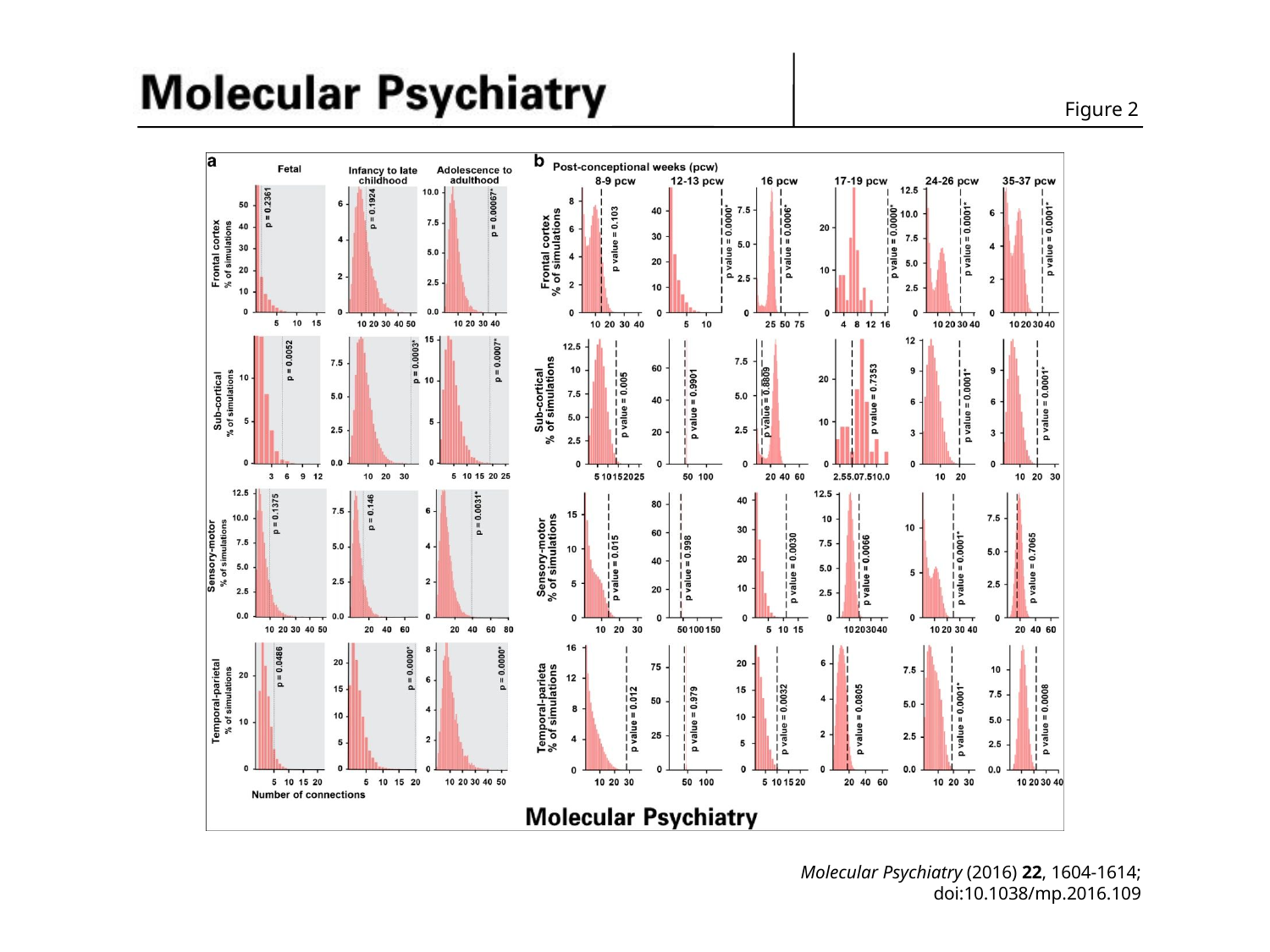

Figure 2
Molecular Psychiatry (2016) 22, 1604-1614;
doi:10.1038/mp.2016.109

Supplement: Supplementary file 8 — PowerPoint slide for Fig. 2 [file 41380_2017_BFmp2016109_MOESM123_ESM.ppt]

## Slide 1
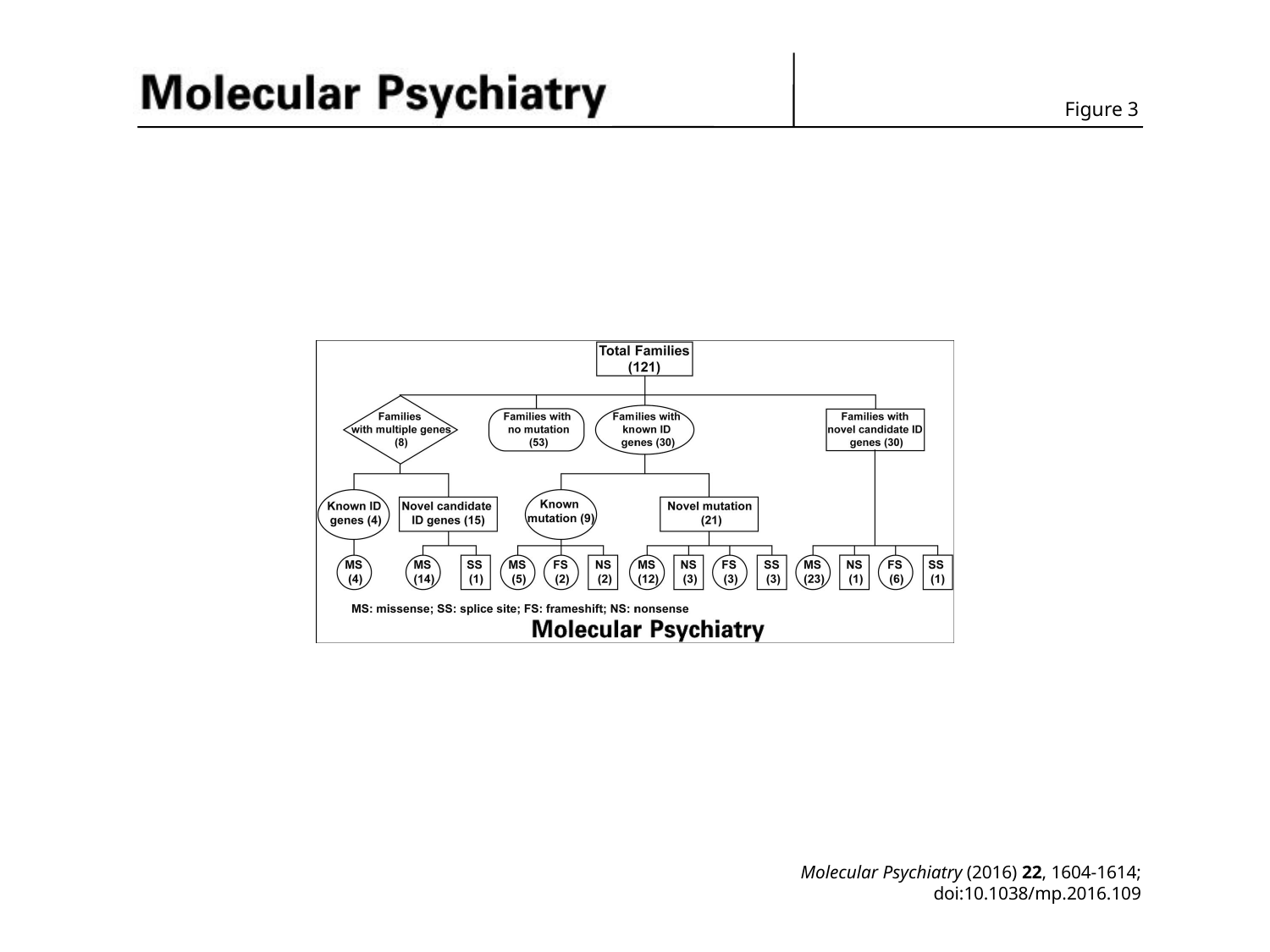

Figure 3
Molecular Psychiatry (2016) 22, 1604-1614;
doi:10.1038/mp.2016.109

Supplement: Supplementary file 9 — PowerPoint slide for Fig. 3 [file 41380_2017_BFmp2016109_MOESM124_ESM.ppt]
